# Circ0515 reprogramming mitochondrial succinate metabolism and promotes lung adenocarcinoma progression through regulating SDHB

**DOI:** 10.1038/s41419-025-07830-7

**Published:** 2025-07-05

**Authors:** Yixiao Yuan, Yue Wu, Chunhong Li, Zuotian Huang, Dadi Peng, Zhongjun Wu, Xiulin Jiang

**Affiliations:** 1 The First Affiliated Hospital of Chongqing Medical University, Chongqing, China; 2 Department of Medicine, UF Health Cancer Center, University of Florida, Gainesville, FL USA; 3 Department of Oncology Suining Central Hospital, Suining, Sichuan China; 4 Chongqing University Cancer Hospital, Chongqing, China

**Keywords:** Cancer epigenetics, Diagnostic markers

## Abstract

Non-small cell lung cancer (NSCLC) is one of the leading causes of cancer-related mortality worldwide. Its high incidence and poor prognosis are closely associated with complex molecular mechanisms. Circular RNAs (circRNAs), a class of non-coding RNAs, play significant regulatory roles in tumorigenesis and progression. However, their specific functions and mechanisms in lung cancer remain largely unclear. This study aims to elucidate the expression pattern and molecular mechanisms of circ0515 in lung cancer, particularly its roles in tumor proliferation, migration, and metabolism. The study revealed that circ0515 is significantly upregulated in lung cancer tissues and cell lines, specific knockdown of circ0515 using short hairpin RNA (shRNA) or antisense oligonucleotide (ASO) significantly inhibits lung cancer cell proliferation, migration, and xenograft tumor formation. On one hand, circ0515 acts as a molecular sponge for miRNA-328-3p, upregulating its downstream target gene YWHAZ, thereby activating the AKT signaling pathway and significantly promoting lung cancer cell proliferation and migration. On the other hand, circ0515 recruited RNA binding motif protein 45 (RBM45) to stabilize SDHB mRNA, promoting SDHB expression and mitochondrial oxidative phosphorylation and succinate metabolism, leading to increased cisplatin resistance in lung cancer cells. These findings not only advance our understanding of the functional roles of circ0515 in lung cancer but also provide a theoretical basis for considering circ0515 as a potential therapeutic target for NSCLC.

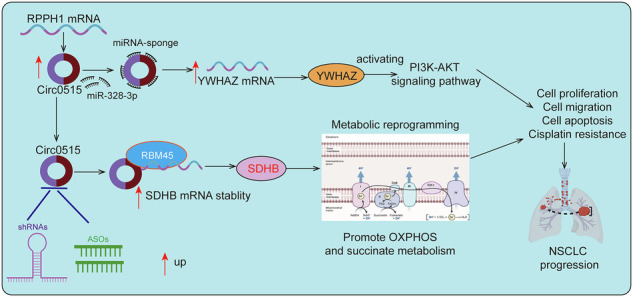

## Introduction

Lung cancer is currently one of the most prevalent malignant tumors worldwide, ranking second in incidence among all malignant tumors globally, but it holds the highest mortality rate [1]. Lung cancer is primarily classified into two major types based on its pathological characteristics: small cell lung cancer (SCLC) and non-small cell lung cancer (NSCLC), with the latter accounting for ~85% of cases [2]. As the most common type of NSCLC, the majority of lung adenocarcinomas are already in the advanced stages of the disease when first diagnosed, leading to the loss of optimal surgical opportunities [3]. Although there have been significant advancements in the early screening and treatment of lung adenocarcinoma in recent years, the 5-year survival rate for patients remains below 20% [4]. This phenomenon is mainly attributed to two factors: first, most patients with lung adenocarcinoma are diagnosed at an advanced stage, missing the optimal timing for surgery; second, during treatment, tumor cells often develop resistance to chemotherapeutic drugs, leading to treatment failure and disease progression [5]. Therefore, exploring the molecular mechanisms underlying the occurrence and development of lung adenocarcinoma, as well as its chemotherapy resistance, and identifying new effective biomarkers, is of great significance for the treatment of lung adenocarcinoma.

In recent years, circular RNAs (circRNAs) have gradually become a focus in the field of malignant tumor research [6]. CircRNAs are a class of non-coding RNAs with a unique closed circular structure, formed by back-splicing of precursor mRNA [7, 8]. They are characterized by diversity, high abundance, high stability, conservation, and tissue specificity [9]. Under normal physiological conditions, the expression of circRNAs is maintained in a balanced state, but under different pathological conditions, their expression profiles undergo significant changes [10]. Increasing evidence suggests that the expression profiles of circRNAs in lung adenocarcinoma tumor tissues change significantly and may be related to the disease’s occurrence and development [11]. Numerous studies have shown that altered circRNA expression profiles are closely associated with various diseases, especially different malignant tumors and the development of chemotherapy resistance [12]. Thus, differentially expressed circRNAs may serve as novel diagnostic and prognostic biomarkers for malignant tumors and may also become new therapeutic targets [13].

Advances in cancer research have revealed many signaling pathways closely associated with tumorigenesis and progression. The phosphatidylinositol-3-kinase/protein kinase B (PI3K/AKT) signaling pathway, as one of the key intracellular signaling pathways, controls important cellular biological processes and cell cycle regulation by affecting the activity of downstream effector molecules involved in apoptosis, transcription, translation, metabolism, and angiogenesis [14–16]. Mutations in certain components of the PI3K/AKT pathway can lead to functional alterations, not only regulating the proliferation and survival of tumor cells but also being closely related to tumor invasion, metastasis, and drug resistance [17, 18]. AKT, the core of the PI3K/AKT signaling pathway, has been found to be upregulated in its activated form (p-AKT) in 43–90% of NSCLC cases, and its expression in early primary tumors is often associated with poor prognosis [19, 20]. However, the role of circRNAs in the regulation of PI3K/AKT signaling pathway activation remains unclear.

Tumor metabolism has been extensively studied in cancer, particularly the ability of tumor cells to rewire and reprogram their metabolic processes to gain growth advantages, survival, and invasive capabilities [21]. For these reasons, metabolic reprogramming is now recognized as one of the “new” hallmarks of cancer. Mitochondria, as dynamic organelles responsible for energy production and cellular metabolism, play a critical role in maintaining tissue integrity. Mitochondrial dysfunction compromises this integrity and is associated with numerous human diseases [22]. Consequently, the concept of “metabolic reprogramming” has gained prominence in cancer research, emphasizing how tumor cells adapt their metabolic pathways for survival and progression [23]. While glycolysis has been extensively studied as a metabolic pathway hijacked by tumor cells, it is not the only pathway exploited. Even metabolic processes traditionally thought to be downregulated in cancer, such as oxidative phosphorylation (OXPHOS), may play significant roles in tumor growth and survival [24]. Indeed, although higher OXPHOS activity is associated with better prognosis in certain cancers and is often downregulated in others, evidence suggests that OXPHOS can be not only utilized but also upregulated in some tumor types [25]. OXPHOS, as the primary mitochondrial metabolic process, provides most of the cellular energy (ATP) [26]. In lung cancer cells, enhanced OXPHOS may facilitate adaptation to changing metabolic demands. Moreover, reactive oxygen species (ROS) generated during OXPHOS play critical roles in cellular signaling and oxidative stress responses, potentially influencing cisplatin-induced DNA damage [27]. However, the role of circular RNAs (circRNAs) in reshaping mitochondrial metabolism remains largely unexplored. Identifying and elucidating the function and mechanism of circRNAs involved in mitochondrial metabolic reprogramming in lung cancer is of paramount importance for advancing diagnostic and therapeutic strategies.

A significant body of research indicates that the circRNA expression profile in NSCLC undergoes notable changes. We also conducted high-throughput microarray dataset analysis of NSCLC tumor tissues and adjacent normal tissues(GSE158695), which revealed 109 differentially expressed circRNAs (including 33 upregulated and 76 downregulated circRNAs) [28]. Among these, hsa_circ_0000515 was found to be the most significantly upregulated in NSCLC, circ_0000515 is derived from the RPPH1 gene. Research has shown that it plays a pro-carcinogenic role in breast cancer and gastric cancer [29, 30]. However, its function in most tumors, particularly lung cancer, has not been previously reported. Therefore, we selected hsa_circ_0000515 as the subject for in-depth research to further explore its role and molecular mechanisms in the progression of lung adenocarcinoma.

In this study, we discovered that hsa_circ_0000515 (hereafter referred to as circ0515 for brevity) is significantly upregulated in lung cancer. High circ0515 expression correlates with shorter overall survival in patients. Circ0515 is predominantly expressed in the cytoplasm of lung cancer cells. Mechanistically, circ0515 functions as a molecular sponge for miR-328-3p, leading to the upregulation of its downstream target gene YWHAZ, which activates the AKT signaling pathway to promote lung cancer cell proliferation and migration. Additionally, circ0515 interacts with RBM45, recruiting this protein to stabilize its downstream target SDHB. This stabilization remodels mitochondrial oxidative phosphorylation metabolism, enhances cisplatin resistance, and accelerates lung cancer progression.

## Materials and method

### Sample collection

We collected cancerous tissues and corresponding adjacent non-cancerous tissues from 25 lung cancer patients who underwent surgical treatment at the First Affiliated Hospital of Chongqing Medical University between May 2017 and December 2022. The tissues were preserved in liquid nitrogen. Inclusion criteria: patients confirmed to have lung cancer through pathological and imaging examinations; first-time diagnosis; no prior treatment before surgery. Exclusion criteria: patients with chronic diseases such as diabetes or hypertension; those with organ dysfunction in the liver, kidneys, etc. Informed consent was obtained from the patients.

### Cell culture, plasmid construction and lentivirus production

Construct independent shRNAs targeting different regions of circ0515 genes using the pLKO.1 vector. The specific shRNA sequences are listed in Table S1. Full-length circ0515 was amplified from 293T cell cDNA and cloned into the PCDH-ZW1-FCS-circRNA vector. All constructs were sequence-verified. Lentivirus production involved transfecting shRNA and overexpression plasmids into 293T cells using PEI transfection reagent, collecting lentivirus at 48 and 72 h post-transfection. Human bronchial epithelial cell line (BEAS-2B) and HEK-293T cells were purchased from ATCC, while BEAS-2B, H1975, H1650, A549, and H1299 cells were from Cobioer, China, with STR documentation. Negative control, miRNA-328-3p, circ0515 ASO and Negative control were purchased from Guangzhou Ruibo Biotechnology and transfected using PEI.

### RNA fluorescence in situ hybridization

The subcellular localization of circ0515 in A549 lung cancer cells was detected using a specific biotin-labeled circ0515 probe. Cells were fixed in 4% paraformaldehyde, permeabilized with 0.5% Triton X-100, and hybridized with the probe at 37 °C overnight in hybridization buffer. After washing to remove unbound probes, the signal was amplified using fluorescently labeled streptavidin and visualized under a confocal microscope. DAPI was used to stain nuclei for reference.

### RNase R digestion assay

Total RNA was extracted from collected cells. A total of 2 μg of RNA was used, with 1 μg as the control group (labeled as RNase R-) and the other 1 μg as the experimental group (labeled as RNase R+). The experimental group was treated with 3U RNase R following the manufacturer’s instructions, with the reaction carried out at 37 °C for 15 min and inactivated at 70 °C for 10 min. The RNase R- control group did not undergo RNase R digestion.

### Actinomycin D treatment assay

A549 and H1975 cells were seeded in 6-well plates at a density of 1 × 10^4^ cells/well and cultured in a serum-free medium for 24 h in a cell incubator. Cells were then treated with 5 μg/mL actinomycin D solution and cultured at different time intervals. Cells were collected, and total RNA was extracted for RT-qPCR analysis.

### RNA isolation, reverse transcription and qRT-PCR

RNA isolation was performed on ice using the Trizol method to extract total RNA from cells with gene knockdown. Depending on the precipitate amount, an appropriate volume of RNase-free water was added to dissolve the RNA. 1 μg of total RNA transcription to obtain cDNA using the Vazyme HiScript IV RT SuperMix for qPCR (R423-01) in an RNase-free environment. Each sample was run in triplicate under the conditions specified in the manual. β-actin as the internal reference gene. The expression levels of circ0515, ACTIN, YWHAZ, EIF4EBP1, ITGA5, and PPP2R5D were calculated using the 2^−ΔΔCt^ method. Specific primer sequences are detailed in Table S1.

### Colony formation assay

A549 and H1975 cells were seeded into 6-well plates at 500 cells per well and cultured for 9–12 days under the same conditions. The colonies were fixed with 4% PFA for 20 min, stained with crystal violet for 20 min washed with water, and then photographed for colony counting.

### CCK-8 assay

Cells in the logarithmic growth phase were collected from each group, digested with trypsin, and centrifuged to obtain cell pellets, which were then resuspended in complete medium. Cells were seeded at a density of 2 × 10^3^ cells/well in 96-well plates and cultured in an incubator. After 24 h, 48 h, and 72 h of culture, the original medium in each well was discarded, and 100 μL of reaction solution (CCK-8 reagent: complete medium = 1:9) was added to each well under aseptic conditions. A blank control well was also set up. The plates were incubated for 1 h, and absorbance was measured at 450 nm.

### Cell apoptosis assay

A549 and H1975 cells with gene knockdown were seeded into 6-well plates and cultured to 75% confluence. Cells were collected, washed and incubated with Annexin V-FITC and PI (Vazyme- A211-01/02) staining solution in the dark for 10–15 min. Apoptotic cells were analyzed using flow cytometry analysis.

### Western blot

A549 and H1975 cells with gene knockdown were collected and washed with cold PBS. RIPA lysed on ice for 30 min. After centrifugation, the supernatant obtained the protein samples were subjected to SDS-PAGE. The membrane was washed with TBST and developed using ECL substrate. The results were captured using an imaging system. The antibodies are shown in Table S2.

### PAR-CLIP for identifying RBM45 targets

PAR-CLIP was performed to identify RBM45 target RNAs in A549 cells. Cells were incubated with 4-thiouridine (4SU) to label nascent RNA, followed by UV crosslinking at 365 nm to induce protein-RNA crosslinks. Lysates were prepared and subjected to immunoprecipitation using an anti-RBM45 antibody. RNA-protein complexes were digested with RNase to generate RNA fragments, which were extracted, reverse-transcribed, and sequenced. High-throughput sequencing data were analyzed to map RBM45-binding sites and identify target RNAs.

### RNA pull down

A549 lung cancer cells were lysed in RIP buffer containing protease and RNase inhibitors. A biotin-labeled circ0515 probe and a control probe were synthesized (Ruibo)and incubated with the lysates at 4 °C overnight. The complexes were captured using streptavidin-coated magnetic beads, followed by thorough washing to remove non-specifically bound molecules. The bound proteins were eluted and identified via mass spectrometry in harvard medical school, taplin mass spectrometry facility, while the associated miRNAs were extracted using TRIzol reagent and analyzed by qRT-PCR.

### RNA immunoprecipitation

A549 lung cancer cells were lysed in RIP lysis buffer supplemented with RNase and protease inhibitors. The lysates were incubated with magnetic beads conjugated to an anti-RBM45 antibody or IgG control at 4°C overnight. After washing, the RNA-protein complexes were eluted, and the bound RNAs were extracted using TRIzol reagent. The enriched RNAs were analyzed by qRT-PCR to identify downstream targets of RBM45.

### Untargeted metabolomics analysis

A549 lung cancer cells transfected with circ0515 shRNA or negative control were collected and lysed in cold methanol:acetonitrile:water (2:2:1, v/v/v) for metabolite extraction. After centrifugation, the supernatants were analyzed using ultra-high-performance liquid chromatography coupled with high-resolution mass spectrometry (UHPLC-HRMS) in UCLA Metabolomics Center. Raw data were processed to identify and quantify metabolites, followed by multivariate statistical analysis to determine differentially expressed metabolites associated with circ0515 knockdown.

### Mitochondrial complex activity and metabolite assays

A549 cells with circ0515 knockdown and control cells were harvested to assess mitochondrial complex I-IV activities and intracellular succinate, fumarate, and ATP levels using commercial kits (Sigma-Aldrich) following the manufacturer’s protocols. Mitochondrial complexes I-IV activities were measured spectrophotometrically, while succinate, fumarate, and ATP levels were quantified via colorimetric or luminescent assays. Data were normalized to total protein content.

### Seahorse energy metabolism analysis

Seahorse energy metabolism analysis was performed based on protocols established in previously published studies [31]. To evaluate glycolytic activity and oxidative phosphorylation levels, the Agilent Seahorse XF Cell Mito Stress Test Kit was used. Cells were seeded into Seahorse XF96 cell culture microplates at a density of 1 × 10⁶ cells/mL in advance. Taking the measurement of extracellular acidification rate (ECAR) as an example, cells were pretreated according to experimental requirements and incubated in Seahorse XF glycolysis stress test medium containing glucose, glutamine, sodium pyruvate, and HEPES buffer. Basal ECAR levels were recorded over three measurement cycles. Glucose, oligomycin (Oligo), and 2-deoxy-D-glucose (2-DG) were subsequently added to monitor real-time changes in glycolytic activity. For oxygen consumption rate (OCR) measurement, a series of compounds including oligomycin and FCCP were sequentially added to assess real-time changes in OCR, reflecting mitochondrial respiration activity.

### Measurement of mitochondrial respiratory chain complex I–V Activities

The activities of mitochondrial respiratory chain complexes I–V were measured using commercially available assay kits according to the manufacturers’ instructions. Briefly, enriched mitochondrial fractions were isolated from cells using the MITOISO2 mitochondrial isolation kit (Sigma-Aldrich, Cat# MITOISO2-1KT). The enzymatic activities of Complex I (NADH dehydrogenase), Complex II (succinate dehydrogenase), Complex III (cytochrome bc1 complex), Complex IV (cytochrome c oxidase), and Complex V (ATP synthase) were then quantified using their respective activity assay kits (Sigma-Aldrich and other suppliers as applicable). For each cell line, 5 × 10⁶ cells were used per assay.

### Dual-luciferase reporter assay

293T cells were cultured in 24-well plates (5 × 10^5^ cells/well) for 24 h and co-transfected with circ0515 WT (or MUT) and miR-328-3p mimic (or miR-NC) using Lipofectamine™ 2000. After 24 h, cells were collected and lysed, and the supernatant was centrifuged at 350 × *g* for 10 min. 20 μL of the supernatant was mixed with 100 μL of firefly luciferase substrate, and luminescence was measured. The luciferase activity of cells was expressed as the ratio of the two.

### KEGG enrichment analysis

To further explore the potential biological functions of miR-328-3p downstream target genes in LUAD, we submitted these genes to the DAVID website for KEGG analysis. The enrichment score (−log10 (*p*-value)) was used to indicate the significance of the correlation between miR-328-3p downstream target genes and the corresponding KEGG pathways in LUAD tumor tissues. The pathways with the highest enrichment scores in the KEGG database were displayed using bubble charts.

### Animal models

A549 lung cancer cells with gene knockdown and control A549 cells were injected subcutaneously into 4–6 week-old female BALB/c nude mice. Tumor growth was monitored by measuring tumor dimensions weekly, and tumor volume was calculated. At the experimental endpoint or humane endpoint criteria, mice were euthanized, tumors were excised under sterile conditions, and tumor weights were measured. A549 cells (1.5 × 10⁶ cells per site) were injected into 4–6 week-old female BALB/c nude mice. When the tumor volume reached 100 mm³, ASO and DDP were administered. ASO was injected intratumorally (5 nM), and DDP was administered intraperitoneally, with injections given every 4 days. All animals were kept in a SPF environment and the protocols were pre-approved and conducted under the policy of Animal care and Use Committee at the the First Affiliated Hospital of Chongqing Medical University.

### Statistical analysis

The expression levels of circ0515 and miR-328-3p in bronchial epithelial cells BEAS2B and lung cancer cell lines (A549, H1299, H1650, H1975) were compared using one-way ANOVA, followed by LSD-t tests for within-group comparisons. For other groups, independent sample t-tests were used for comparison between two groups. The data were analyzed by two-tailed *t* test. The indicated *P* values (*P < 0.05, **P < 0.01 and ***P < 0.001) were considered statistically significant.

## Results

### circ0515 is significantly upregulated in lung cancer tissues

**Fig. 1:**
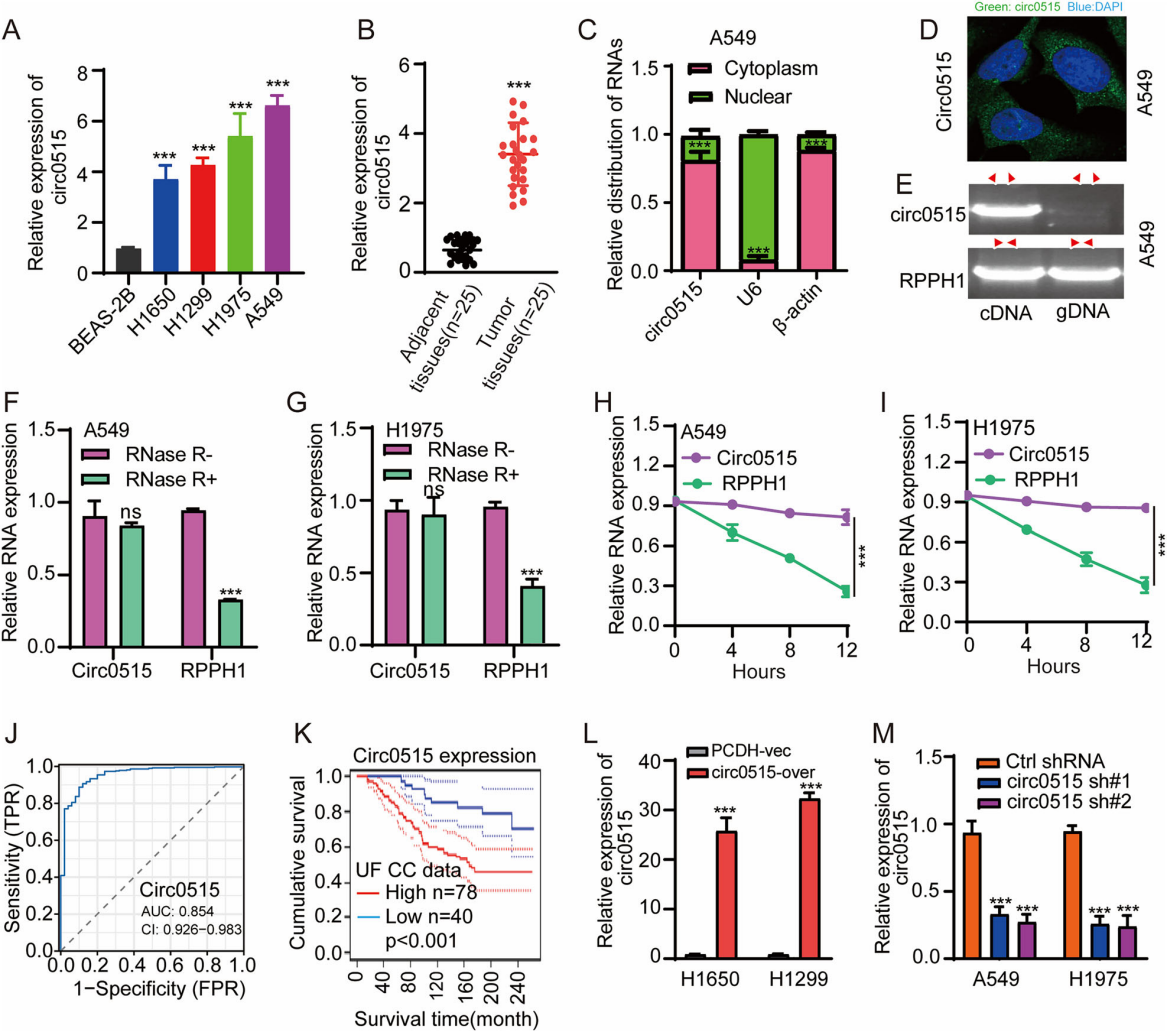
Circ0515 was highly expressed in lung cancer. **A** qPCR experiments were conducted to detect the expression levels of circ0515 in lung cancer cells compared to normal lung epithelial cells. **B** qPCR experiments were performed to assess circ0515 expression levels in 25 pairs of matched lung cancerous and adjacent non-cancerous tissues. **C**, **D** RNA nuclear-cytoplasmic separation and FISH experiments were conducted to determine the subcellular localization of circ0515. **E** The expression of circ0515 was evaluated using gDNA and cDNA as templates. **F**, **G** RNAse R treatment was used to examine the expression of circ0515 and its corresponding parental gene in NSCLC cells lines. **H**, **I** Actinomycin D (5 μg/mL, Sigma) treatment was performed to assess changes in RNA stability of circ0515 and its corresponding parental gene. **J** ROC curve analysis of circ0515 in lung cancer samples collected in this study. **K** Survival analysis of circ0515 expression in lung cancer patients from UF cancer center. **L**, **M** qPCR experiments were performed to assess the efficiency of circ0515 knockdown and overexpression in diverse lung cancer cells. PCDH PCDH-Vec, circ0515 Over circ0515 Overexpression, sh#1 circ0515 shRNA#1, sh#2 circ0515 shRNA#2. ns means not significant, *P < 0.05, **P < 0.01, ***P < 0.001 (Student’s *t*-test).

A significant body of research indicates that the circRNA expression profile in NSCLC undergoes notable changes. We also conducted high-throughput RNA sequencing data analysis (GSE158695) of NSCLC tumor tissues and adjacent normal tissues, which revealed 109 differentially expressed circRNAs (including 33 upregulated and 76 downregulated circRNAs) (Table S3). Among these, circ0515, is derived from the RPPH1 gene, was found to be the most significantly upregulated in NSCLC. However, the function of circ0515 in lung adenocarcinoma remains unclear, and this study aims to investigate its role and underlying mechanisms in LUAD progression. Using qRT-PCR, we observed that circ0515 expression was significantly elevated in most lung cancer cell lines, especially in A549 and H1975, compared to normal lung epithelial cells (Fig. 1A). Additionally, in 25 paired lung cancer and adjacent normal tissue samples, obtained from our hospital, results showed that circ0515 was also significantly upregulated in lung cancer tissues, consistent with the cellular level results (Fig. 1B). RNA nuclear-cytoplasmic separation and FISH experiments revealed that circ0515 is primarily localized in the cytoplasm (Fig. 1C, D). Moreover, PCR results using different primers indicated that circ0515 is amplified from cDNA rather than genomic DNA of lung cancer cells (Fig. 1E). The resistance of circ0515 to RNase R digestion, as shown by actinomycin D and RNase R exonuclease treatments, further supports its high stability as a circular RNA (Fig. 1F–I). ROC curve analysis demonstrated that circ0515 could serve as a potential and effective diagnostic biomarker (Fig. 1J). Data from the University of Florida Cancer Center revealed that lung cancer patients with higher circ0515 expression had significantly shorter overall survival (Fig. 1K). Overall, these results indicate that circ0515 is abnormally expressed in lung cancer and may be involved in the malignant progression of the disease.

### Circ0515 promotes tumor growth in vitro and in vivo

**Fig. 2:**
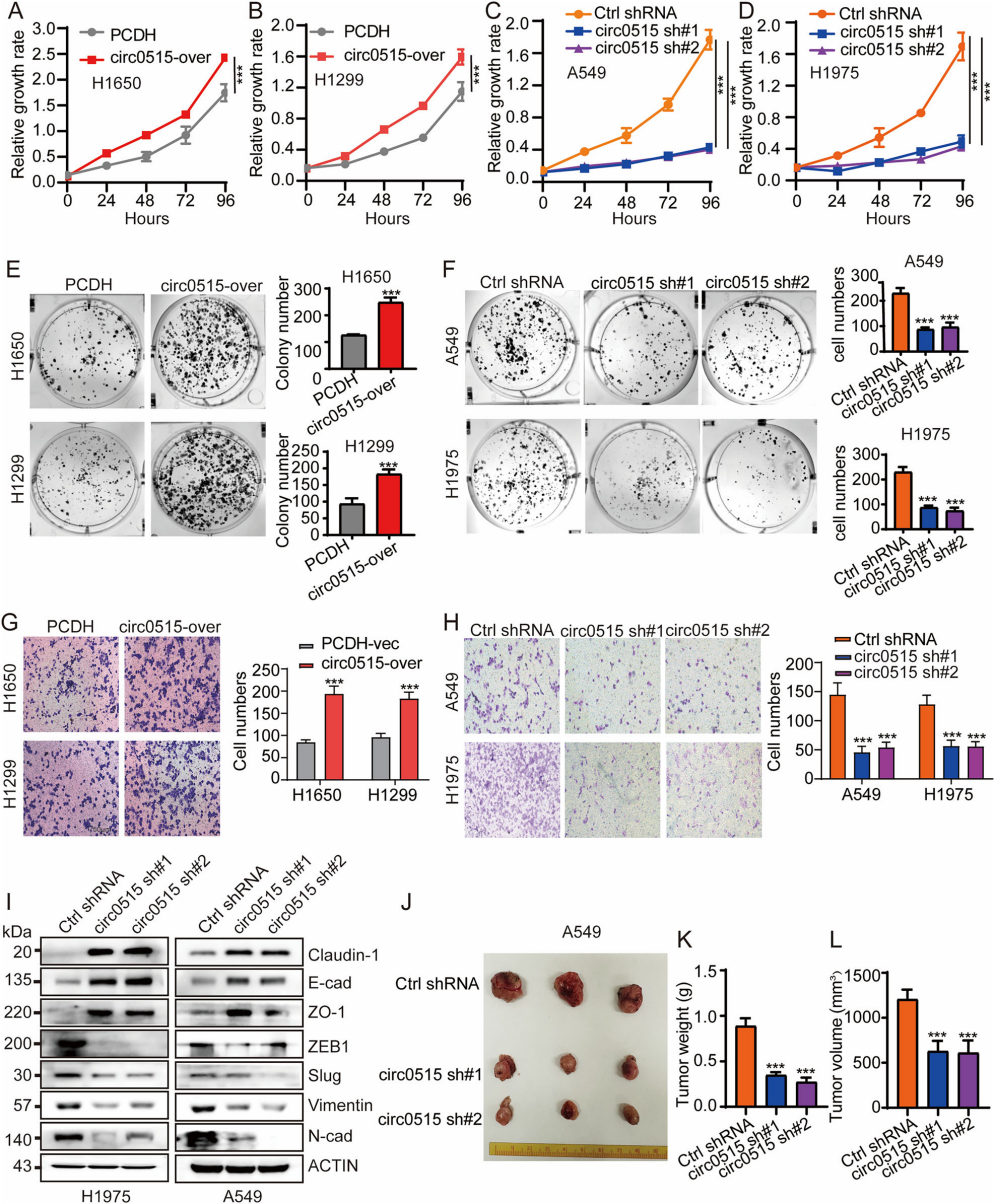
Knockdown of circ0515 inhibits proliferation, cell migration and tumor growth in lung cancer. **A**–**D** CCK8 experiments detect the effect of circ0515 knockdown and overexpression on lung cancer cell proliferation. **E**, **F** Clonogenic assays detect the effect of circ0515 knockdown and overexpression on lung cancer cell colony formation ability. **G**, **H** Transwell assays detect the effect of circ0515 knockdown and overexpression on lung cancer cell migration ability. **I** Western blot experiments detect the effect of circ0515 knockdown on EMT-related proteins in lung cancer cells. **J**–**L** Xenograft experiments in nude mice to assess the impact of the circ0515 knockdown on lung cancer tumor growth. **K** shows the tumor weight, **L** shows the tumor volume, (*n* = 3 mice per group), PCDH PCDH-Vec, circ0515 Over circ0515 Overexpression, sh#1 circ0515 shRNA#1, sh#2 circ0515 shRNA#2. NC Negative control, mimics miR-328-3p mimics, ns means not significant, *P < 0.05, **P < 0.01, ***P < 0.001 (Student’s *t*-test).

Given the differential expression of circ0515 in various lung cancer cell lines, we selected A549 and H1975 cells, which exhibit the highest expression levels, to construct circ0515 knockdown cell lines, and H1650 and H1299 cells, with lower expression levels, to construct circ0515 overexpression cell lines. The knockdown and overexpression efficiencies were verified by qPCR (Fig. 1L, M), and the effects of circ0515 on the malignant phenotype of lung cancer cells were further investigated. To further explore the functional role of circ0515 in lung cancer progression, we performed CCK8 and colony formation assays, which revealed that overexpression of circ0515 enhanced the proliferative and growth capacities of lung cancer cells, while knockdown of circ0515 significantly inhibited these abilities (Fig. 2A–F). Wound healing assays demonstrated that circ0515 overexpression enhanced lung cancer cell migration compared to controls, while knockdown inhibited migration (Fig. S1A–S1C). Additionally, Transwell assays showed that circ0515 overexpression promoted lung cancer cell invasion, with knockdown producing the opposite effect (Fig. 2G, H). We also examined the expression of EMT-related proteins in response to circ0515 knockdown, finding that it upregulated Claudin-1, E-cadherin, and ZO-1, while downregulating ZEB1, Slug, Vimentin, and N-cadherin (Fig. 2I and Fig. S1D–1E). In vivo, using a mouse xenograft tumor model, we found that circ0515 knockdown inhibited lung cancer tumor growth, including tumor size and volume, a result consistent across two lung cancer cell lines(Fig. 2J–L and Fig. S1F–S1H). These findings indicate that high circ0515 expression promotes lung cancer growth.

### miR-328-3p suppresses lung adenocarcinoma progression

Since circRNA localization is often related to its functional mechanism, cytoplasmic circRNAs generally act as miRNA sponges. We further analyzed miRNAs differentially expressed in TCGA-LUAD (Fig. S2A), using CircBank, we predicted miRNAs that interact with circ0515. The results from two datasets were intersected to identify common miRNAs, including hsa-miR-326, miR-328-3p, and hsa-miR-1976, that potentially interact with circ0515 (Fig. S2B). To further identify the miRNAs interacting with circ0515, we performed RNA pull-down experiments using biotin-labeled probes for circ0515. The results revealed that miR-328-3p, rather than hsa-miR-1976 and hsa-miR-326, could be pulled down by the probe (Fig. S2C). Therefore, miR-328-3p was selected for further investigation regarding its interaction with circ0515. Subsequent analysis revealed significant downregulation of miR-328-3p in lung cancer tissues as examined by TCGA-LUAD (Fig. S2D). qPCR results from lung cancer adjacent tissues and tumor samples obtained from our hospital showed that miR-328-3p expression was significantly reduced in lung cancer tissues (Fig. S2E). Further analysis confirmed that miR-328-3p was significantly downregulated in lung cancer cell lines, which was also corroborated by clinical sample results and TCGA-LUAD database analysis (Fig. S2F). Additionally, miR-328-3p lower expression was significantly associated with poor prognosis in lung cancer patients (Fig. S2G). Dual-luciferase reporter assays confirmed the interaction between circ0515 and miR-328-3p (Fig. S2H). These findings suggest that circ0515 is upregulated in both lung cancer cell lines and tissues and can interact with miR-328-3p.

**Fig. 3:**
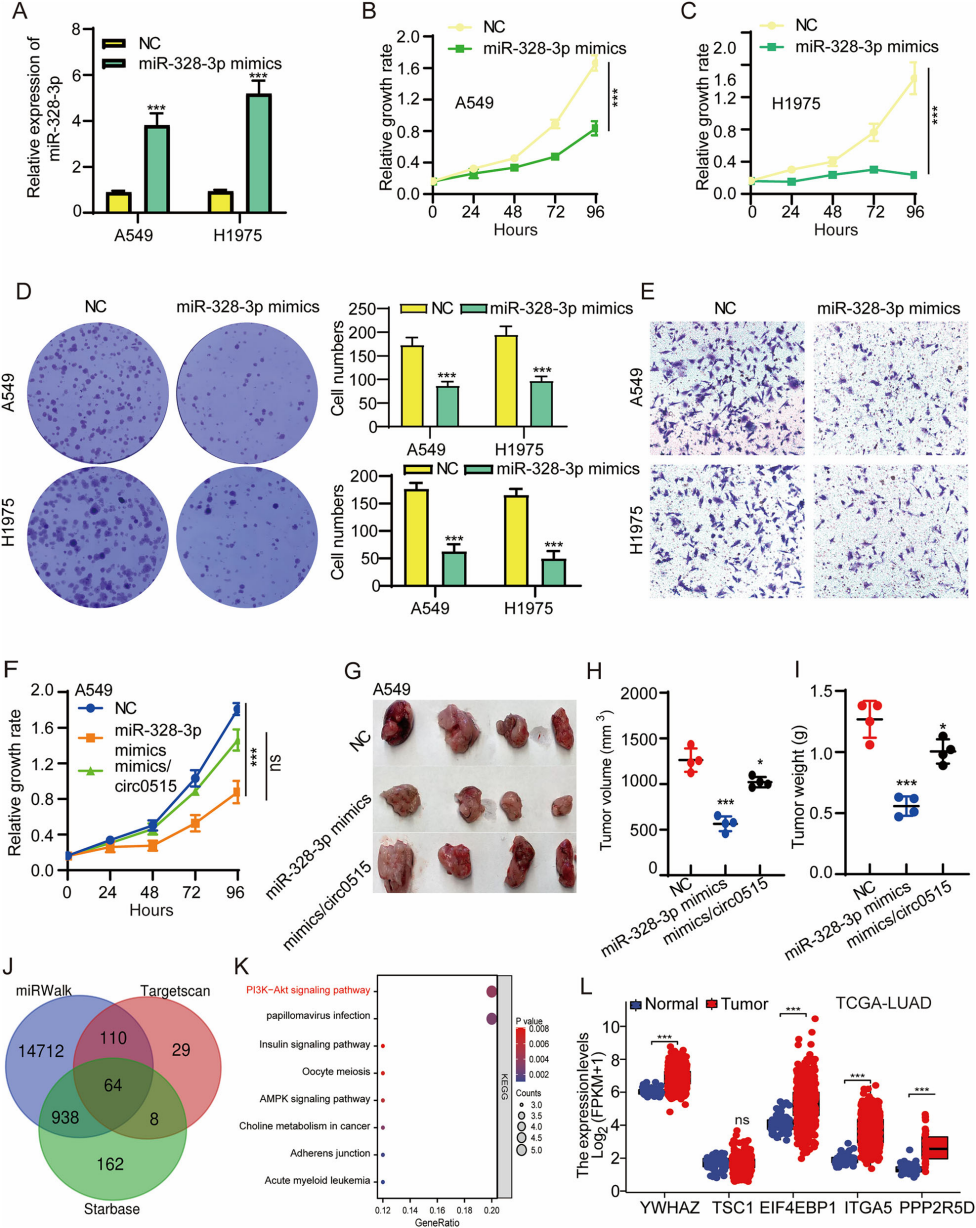
overexpression of miR-328-3p inhibited lung cancer progression. **A** qPCR experiments were performed to assess the efficiency miR-328-3p overexpression in A549 and H1975 lung cancer cells. **B**, **C** CCK8 experiments detect the effect of miR-328-3p overexpression on lung cancer cell proliferation. **D** Clonogenic assays detect the effect of miR-328-3p overexpression on lung cancer cell colony formation ability. **E** Transwell assays detect the effect of miR-328-3p overexpression on lung cancer cell migration ability. **F** Overexpression of miR-328-3p can reverse the promotive effect of circ0515 overexpression on the proliferative capacity of lung cancer cells. **G**–**I** Overexpression of miR-328-3p can reverse the promotive effect of circ0515 overexpression on tumor growth. **H** shows tumor volume, and **I** shows tumor weight (*n* = 4 mice per group). **J** The downstream target genes of miR-328-3p were predicted and identified by intersecting the results from the miRWalk, TargetScan, and starBase databases. **K** Pathway enrichment analysis was performed for the downstream target genes of miR-328-3p. **L** The expression levels of the downstream target genes of miR-328-3p were analyzed using the TCGA-LUAD dataset. PCDH PCDH-Vec, circ0515 Over circ0515 Overexpression, sh#1 circ0515 shRNA#1, sh#2 circ0515 shRNA#2. NC negative control, mimics miR-328-3p mimics, All data and error bars are presented as the mean ± SDs. ns means not significant, *P < 0.05, **P < 0.01, ***P < 0.001 (Student’s *t*-test).

The role of miR-328-3p in lung cancer has not been previously reported. We synthesized miR-328-3p mimics and transfected them into lung cancer cell lines. qPCR validation confirmed successful overexpression of miR-328-3p (Fig. 3A). CCK8 and colony formation assays revealed that miR-328-3p overexpression significantly inhibited lung cancer cell proliferation and growth (Fig. 3B–D). Furthermore, Transwell assays showed that miR-328-3p overexpression significantly inhibited lung cancer cell migration (Fig. 3E). Since circ0515 has been validated as a molecular sponge for miR-328-3p, we further conducted rescue experiments to confirm that miR-328-3p exerts its tumor-suppressive function by targeting circ0515. Our findings revealed that overexpression of circ0515 can abolish the inhibitory effects of miR-328-3p mimics on lung cancer cell proliferation and tumor growth (Fig. 3F–I). These data collectively indicate that miR-328-3p exerts its tumor-suppressive effects by targeting and inhibiting circ0515.

### YWHAZ is a functional target of miR-328-3p in lung cancer cells

**Fig. 4:**
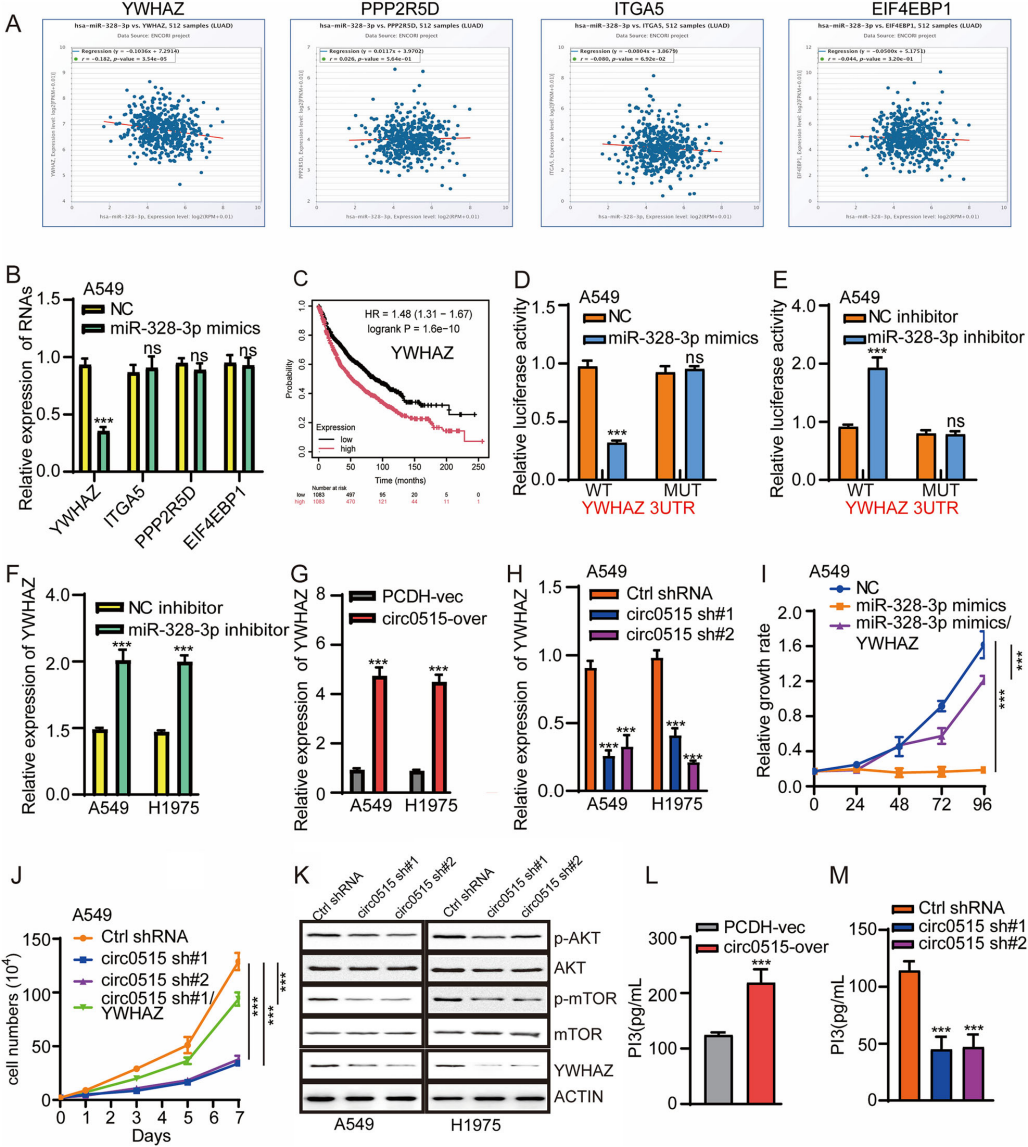
circ0515 activiting the PI3K-AKT signaling pathway. **A** Analysis of the correlation between miR-328-3p and its target gene expression in lung adenocarcinoma using the TCGA database. **B** qPCR analysis of changes in downstream target gene expression following miR-328-3p overexpression. **C** Evaluation of the prognostic value of miR-328-3p target genes in LUAD using the TCGA database. **D**, **E** Dual-luciferase reporter assays to assess the binding interaction between miR-328-3p and its target genes in A549 cells. **F** qPCR analysis of changes in downstream target gene expression following inhibition of endogenous miR-328-3p. **G**, **H** qPCR experiments to determine the expression of miR-328-3p target genes after overexpression or knockdown of circ0515 in lung cancer cells. **I**, **J** Cell proliferation rescue assays involving circ0515/miR-328-3p/YWHAZ interactions. **K** Western blot experiments to detect the effect of circ0515 knockdown on PI3K-AKT signaling pathway-related proteins in lung cancer cells. **L**, **M** ELISA experiments to measure PI3K production following circ0515 knockdown or overexpression in lung cancer cells. PCDH PCDH-Vector control, circ0515 Over circ0515 overexpression, sh#1 circ0515 shRNA#1, sh#2 circ0515 shRNA#2. YWHAZ YWHAZ overexpression. ns means not significant, *P < 0.05, **P < 0.01, ***P < 0.001 (Student’s *t*-test).

To further investigate the downstream targets of miR-328-3p, we used TargetScan, StarBase, and miRWalk databases to predict potential targets of miR-328-3p, identifying 64 candidates (Fig. 3J). KEGG enrichment analysis of these targets revealed their involvement in pathways such as PI3K-AKT and adherens junctions (Fig. 3K). Among these, the PI3K-AKT pathway was the most significantly enriched and critically important in lung cancer progression [32]. We identified five target genes within the PI3K-AKT pathway and found that, except for TSC1, YWHAZ, EIF4EBP1, ITGA5, and PPP2R5D were significantly upregulated in LUAD (Fig. 3L). The TCGA-LUAD expression correlation analysis indicated that YWHAZ, rather than TSC1, EIF4EBP1, ITGA5, and PPP2R5D, was significantly negatively correlated with miR-328-3p (Fig. 4A). To further confirm the downstream target genes of miR-328-3p, we transfected A549 cells with miR-328-3p mimics. The results showed that only YWHAZ was significantly downregulated, while the other four target genes remained unchanged (Fig. 4B). Additionally, higher YWHAZ expression was significantly correlated with poor prognosis in lung cancer patients (Fig. 4C). These results preliminarily suggest that miR-328-3p may negatively regulate the expression of YWHAZ in lung cancer. Further dual-luciferase reporter assays confirmed the interaction between miR-328-3p and YWHAZ (Fig. 4D, E). qPCR results showed that inhibiting endogenous miR-328-3p could upregulate YWHAZ expression levels (Fig. 4F). To investigate whether circ0515 regulates these target genes through miR-328-3p, we overexpressed and knocked down circ0515 in A549 and H1975 cell lines and used qPCR to assess changes in these target genes. Results showed that circ0515 overexpression increased the expression of target genes, while knockdown significantly reduced their expression (Fig. 4G, H). Further rescue experiments confirmed that overexpression of circ0515 promoted lung cancer cell proliferation, an effect that could be abolished by overexpressing miR-328-3p. Conversely, knockdown of circ0515 inhibited lung cancer cell proliferation, an effect that could be rescued by overexpressing YWHAZ (Fig. 4I, J). Previous studies have reported that YWHAZ, as a member of the 14-3-3 protein family, participates in regulating various cellular processes, including signal transduction, cell cycle regulation, apoptosis, and drug resistance [33]. YWHAZ modulates the activation of the AKT signaling pathway by interacting with several upstream molecules, thus enhancing AKT pathway activity and promoting tumor cell proliferation and survival [34]. To further validate the above findings, we used Western blotting to detect the expression of AKT signaling pathway-related proteins. Immunoblotting confirmed that circ0515 knockdown significantly downregulated YWHAZ expression and inhibited the phosphorylation of PI3K/AKT signaling downstream effectors, including Akt and mTOR, compared to total protein levels (Fig. 4K and Fig. S3A, S3B). Additionally, ELISA assays showed that circ0515 overexpression significantly increased, while circ0515 knockdown significantly reduced PIP3 production (Fig. 4L, M). Rescue experiments using SC79, a well-known AKT activator, demonstrated that SC79 could reverse the inhibitory effects of circ0515 knockdown on lung cancer cell proliferation and migration (Fig. S3C, S3D). These findings suggest that circ0515 regulates the abundance of YWHAZ in the PI3K-AKT signaling pathway by acting as a molecular sponge for miR-328-3p.

### Circ0515 binds to RBM45 in lung cancer cells

**Fig. 5:**
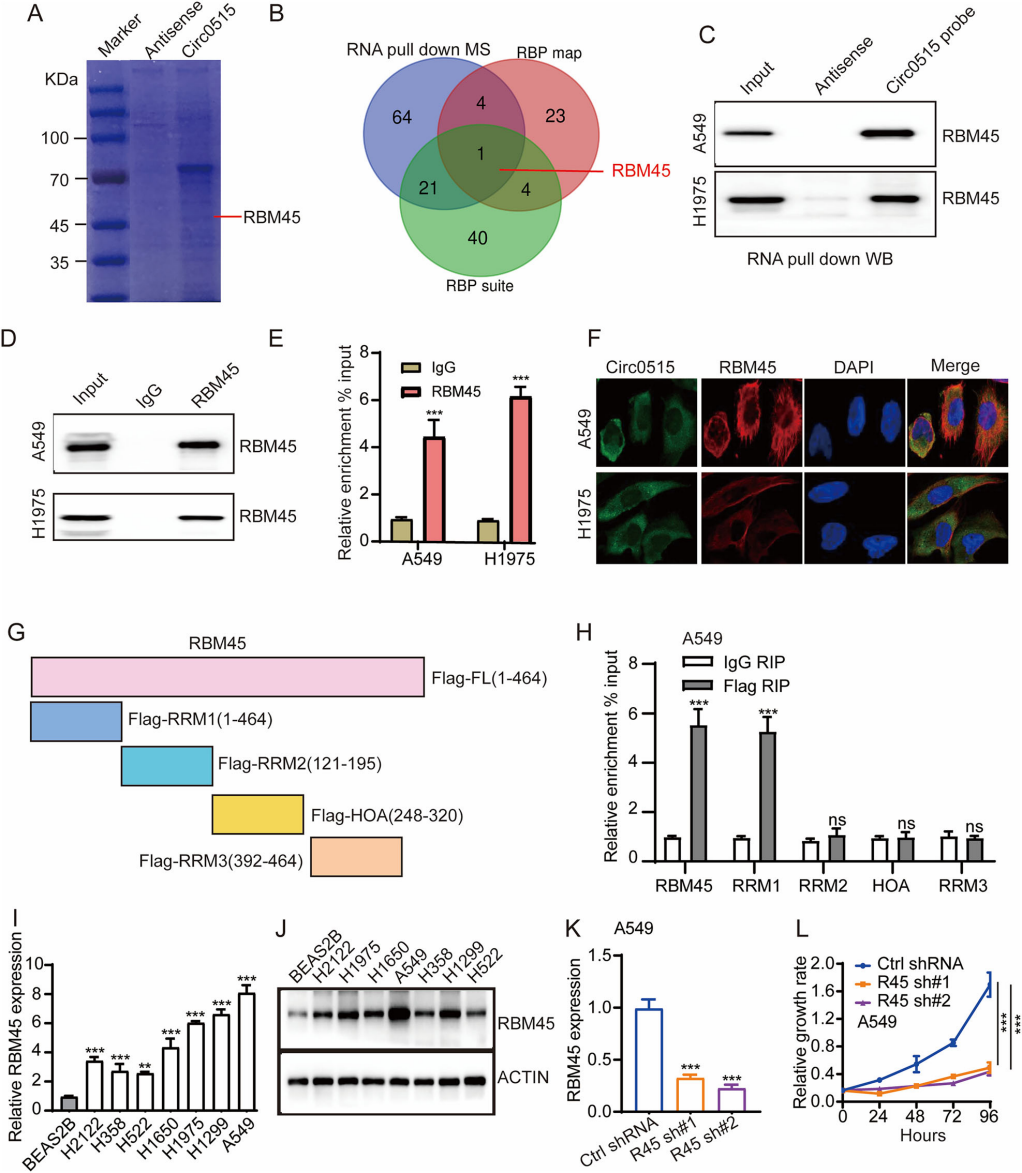
Circ0515 binds to RBM45 in lung cancer cells. **A** Coomassie blue staining of proteins from circ0515 RNA pull-down followed by western blotting. **B** Identification of circ0515-interacting proteins through overlapping results from mass spectrometry and bioinformatic predictions. **C** Validation of circ0515 interaction with RBM45 via RNA pull-down followed by western blotting. **D**, **E** RBM45 RIP assay validating the interaction with circ0515. **F** Immunofluorescence showing colocalization of RBM45 and circ0515 in the cytoplasm. **G**, **H** Truncation experiments identifying RBM45 binding regions with circ0515. **I**, **J** qPCR and Western blot analysis of RBM45 expression in lung cancer cells versus normal lung epithelial cells. **K** qPCR analysis of RBM45 knockdown efficiency in A549 cells. **L** Western blot analysis of RBM45 knockdown efficiency in lung cancer cells. R45 sh#1 RBM45 shRNA#1, R45 sh#2 RBM45 shRNA#2, ns means not significant, *P < 0.05, **P < 0.01, ***P < 0.001 (Student’s *t*-test).

Considering that circ0515 is primarily located in the cytoplasm, it can interact not only with miRNAs but also with proteins. To further explore the functional protein interactions of circ0515, we used a biotin-labeled antisense probe of circ0515 as a control and performed RNA pull-down experiments in A549 cells with a circ0515 probe. The samples were subjected to coomassie blue staining and subsequent proteomic analysis (Fig. 5A). The mass spectrometry results identified 90 potential proteins interacting with circ0515. To refine the identification of circ0515-interacting proteins, we intersected the mass spectrometry results with bioinformatics database predictions (including RBP map and RBP suite). This approach led to the identification of RBM45, a well-known RNA-binding protein that plays a crucial role in regulating RNA stability and protein translation, as a potential interactor with circ0515 (Fig. 5B, C). RIP experiments also confirmed that RBM45 can bind to circ0515 (Fig. 5D, E). Immunofluorescence co-localization experiments demonstrated that circ0515 and RBM45 co-localize in the cytoplasm (Fig. 5F), suggesting that their interaction may play a significant role. Given the complex structure of RBM45, we created truncations of the functional domains of RBM45. The results showed that the RRM1 domain of RBM45, a well-known RNA-binding domain, is capable of interacting with circRNA, indicating the importance of this domain in the interaction with circ0515 (Fig. 5G, H). Although the function of RBM45 in lung cancer has not been reported, our qPCR and Western blot experiments revealed that RBM45 expression is significantly upregulated in lung cancer cell lines (Fig. 5I, J and Fig. S4A). Inhibiting its expression significantly suppressed lung cancer cell proliferation (Fig. 5K, L), Analysis of The Cancer Genome Atlas (TCGA) database revealed that RBM45 is significantly upregulated in lung cancer (Fig. S4B). To further validate this result, we collected clinical samples and performed immunohistochemistry (IHC) experiments. The findings confirmed that RBM45 expression is markedly elevated in lung cancer tissues compared to control tissues (Fig. S4C). Notably, lung cancer patients with high RBM45 expression exhibited significantly shorter overall survival compared to those with low expression (Fig. S4D). Interestingly, receiver operating characteristic (ROC) curve analysis demonstrated that RBM45 has potential diagnostic value for lung cancer (Fig. S4E).

These results suggesting that the interaction between circ0515 and RBM45 may play an important role in lung cancer progression.

### Circ0515/RBM45 controls the transcript stability of mitochondrial respiratory complex proteins

**Fig. 6:**
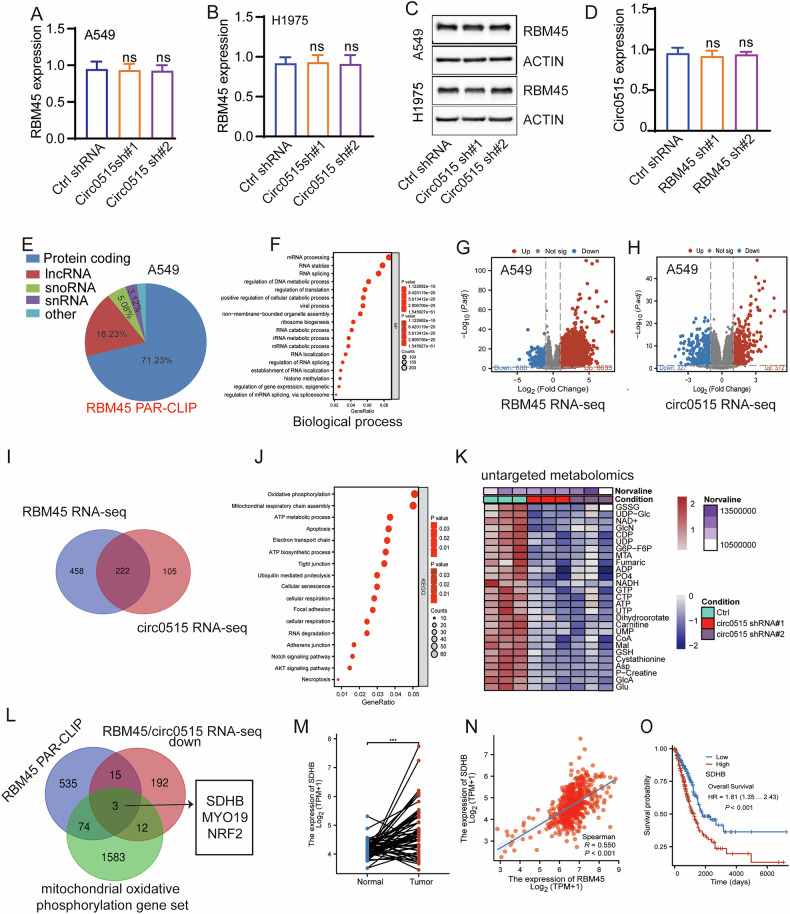
Circ0515/RBM45 controls the transcript stability of mitochondrial respiratory complex proteins. **A**–**C** qPCR and Western blot analysis of the effects of circ0515 knockdown on RBM45 expression in A549 cells. **D** qPCR analysis of the effects of RBM45 knockdown on circ0515 expression in A549 cells. **E** Pie chart analysis of gene types bound by RBM45 from PAR-CLIP data. **F** GO biological process enrichment analysis of RBM45 target genes. **G**, **H** Analysis of differentially expressed genes from RNA-seq after circ0515 or RBM45 knockdown (LogFC < 1, *p* < 0.01). **I** Intersection of differentially expressed genes from RNA-seq data for circ0515 and RBM45 knockdown. **J** KEGG enrichment analysis of circ0515/RBM45 target genes. **K** Untargeted metabolomics results in circ0515 knockdown cell lines. **L** Multi-omics intersection identifying circ0515/RBM45 co-regulated target genes. **M** Analysis of SDHB expression in lung cancer using the TCGA database. **N** Correlation analysis of RBM45 and SDHB expression in LUAD using the TCGA database. **O** Prognostic value of SDHB in LUAD evaluated using the TCGA database. UP up-regulation, down down regulation. PCDH PCDH-Vector control, circ0515 Over circ0515 overexpression, sh#1 circ0515 shRNA#1, sh#2 circ0515 shRNA#2. ns means not significant, *P < 0.05, **P < 0.01, ***P < 0.001 (Student’s *t*-test).

To further investigate the regulatory relationship between circ0515 and RBM45, we found that knockdown of circ0515 did not affect the RNA and protein levels of RBM45, despite their interaction (Fig. 6A–C). Similarly, knockdown of RBM45 did not regulate the expression level of circ0515 (Fig. 6D), suggesting that the binding between circ0515 and RBM45 may be synergistic rather than mutually regulating each other’s expression. Since RBM45 is a classic RNA-binding protein, we first used PAR-CLIP to identify the target genes bound by RBM45. These target genes include lncRNAs, mRNAs, and snRNAs (Fig. 6E), and gene functional enrichment analysis revealed that these targets are primarily involved in RNA stability maintenance and RNA metabolism processes (Fig. 6F). To further clarify the target genes co-regulated by circ0515 and RBM45, we performed RNA sequencing on A549 lung cancer cells with knockdown of circ0515 and RBM45, identifying 680 and 327 significantly downregulated differential target genes (LogFC < 1, *p* < 0.001), respectively (Fig. 6G, H). Due to the synergistic effect, we intersected these downregulated target genes and identified 222 genes co-regulated by circ0515 and RBM45 (Fig. 6I). Functional enrichment analysis of these genes revealed that they are primarily involved in oxidative phosphorylation, mitochondrial electron transport chain, ATP production, and apoptosis pathways (Fig. 6J). Since most of the pathways are focused on mitochondrial-related metabolism, we conducted untargeted metabolomics analysis on A549 lung cancer cells with circ0515 knockdown. The results showed that circ0515 primarily affects mitochondrial function-related metabolism (Fig. 6K), suggesting that circ0515 knockdown partially affects the mitochondrial respiratory chain function, leading to significant changes in downstream metabolites.

To further explore the downstream target genes regulated by circ0515 and RBM45 related to mitochondrial metabolism, we intersected the co-downregulated target genes by circ0515/RBM45 knockdown, the target genes bound by RBM45 in PAR-CLIP, and a mitochondrial oxidative metabolism gene set. This resulted in three potential target genes: SDHB, MYO19, and NRF2 (Fig. 6L). Among these genes, SDHB, which encodes a subunit of mitochondrial complex II, drew significant attention due to its core function in the mitochondrial respiratory chain [35]. Further analysis revealed that SDHB is significantly upregulated in lung cancer and that RBM45 expression is significantly positively correlated with SDHB expression in lung cancer (Fig. 6M, N). Moreover, SDHB expression is negatively correlated with the prognosis of lung cancer patients, and ROC curve analysis suggested that SDHB can effectively differentiate normal from lung cancer patients (Fig. 6O and Fig. S5A). These results suggest the importance of SDHB in lung cancer progression. Given that SDHB is a target gene co-regulated by circ0515 and RBM45, we hypothesize that circ0515 may enhance RBM45 binding to SDHB, thereby stabilizing the transcript of SDHB. To test this hypothesis, we performed RIP experiments, which showed that knockdown of circ0515 indeed reduces the binding of RBM45 to SDHB transcripts, while overexpression of circ0515 enhances RBM45 binding to SDHB (Fig. S5B, S5C). Both circ0515 and RBM45 knockdown decreased SDHB RNA, suggesting that circ0515 and RBM45 may regulate SDHB expression at the post-transcriptional level (Fig. S5D, S5E). Further experiments using actinomycin D, a well-known transcription inhibitor [36], showed that knockdown of circ0515 and RBM45 significantly reduced the transcript stability of SDHB and downregulated its protein levels (Fig. S5F–S5J).

### Circ0515/RBM45/SDHB axis controls succinate metabolism in lung cancer cells

**Fig. 7:**
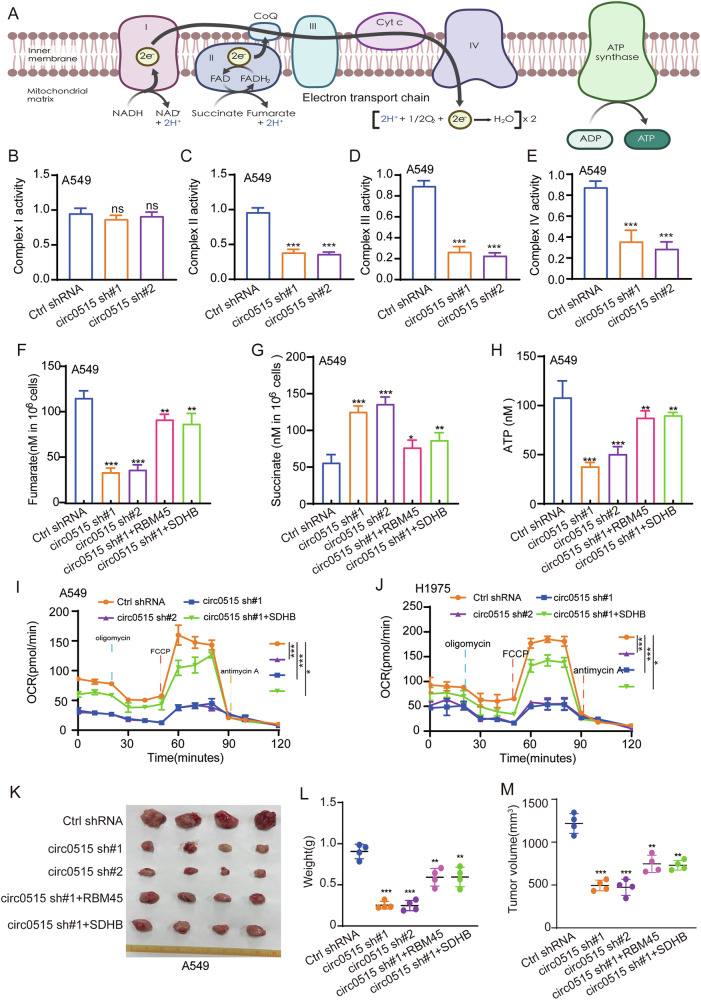
Circ0515/RBM45/SDHB controls succinate metabolism in lung cancer cells. **A** Schematic representation of the mitochondrial respiratory chain complexes. **B**, **E** Analysis of the effects of circ0515 knockdown on the activity of mitochondrial respiratory complexes I-IV using commercial kits. **F**–**H** Effects of circ0515 knockdown on mitochondrial metabolites using commercial kits. **I**, **J** Measure the cellular oxygen consumption rate (OCR) of lung cancer cells after knockdown of circ0515 using Seahorse XF analysis. **K**–**M** Xenograft experiments in nude mice assessing the impact of the circ0515/RBM45/SDHB signaling axis on tumor growth. **L** Tumor weight; **M** Tumor volume, (*n* = 4 mice per group). PCDH PCDH-Vector control, circ0515 Over circ0515 overexpression, sh#1 circ0515 shRNA#1, sh#2 circ0515 shRNA#2. All data and error bars are presented as the mean ± SDs. *P < 0.05, **P < 0.01, ***P < 0.001 (Student’s *t*-test).

Given the critical role of SDHB in the mitochondrial complex, we further investigated whether the circ0515/RBM45/SDHB axis regulates the activity of the mitochondrial complex and its associated metabolism. The mitochondrial respiratory chain consists of four complexes: complexes I, II, III, and IV (Fig. 7A) [37]. As a subunit of complex II, SDHB prompted us to further explore the role of circ0515 and RBM45 in the regulation of the mitochondrial respiratory chain. We found that knockdown of circ0515 significantly downregulated the activity of complexes II, III, and IV of the respiratory chain, but did not affect complex I activity (Fig. 7B–E). Complex II primarily catalyzes the conversion of succinate to fumarate. We further examined the changes in succinate, fumarate, and ATP levels and found that knockdown of circ0515 led to increased succinate, decreased fumarate, and significantly lower mitochondrial ATP levels (Fig. 7F–H). We next quantified mitochondrial respiration of NSCLC cells by measuring oxygen consumption rate (OCR). Compared with Ctrl shRNA group, circ0515 knockdown obviously decreased the OCR level, which was substantially rescued with SDHB overexpression (Fig. 7I, J). Cell proliferation assays showed that the inhibitory effects of circ0515 knockdown on lung cancer cell proliferation could be rescued by overexpression of RBM45 and SDHB, respectively (Fig. S6A–S6B). Similarly, the inhibitory effects of RBM45 knockdown on lung cancer cell proliferation could be rescued by overexpression of circ0515 (Fig. S6C). These results indicate that circ0515 and RBM45 play a synergistic key role in maintaining SDHB stability and lung cancer cell proliferation. To determine whether this axis influences lung cancer tumor growth, in vivo tumor implantation experiments revealed that knockdown of circ0515 and RBM45 suppressed tumor growth, and overexpression of SDHB rescued the inhibitory effects of circ0515 knockdown on tumor growth (Fig. 7K–M). These results suggest that the interaction between circ0515 and RBM45 enhances RBM45 binding to SDHB transcripts, stabilizing SDHB expression and increasing mitochondrial respiratory chain function, promoting mitochondrial metabolism, and facilitating malignant proliferation and metastasis of lung cancer cells.

### Targeting circ0515 to reverse cisplatin resistance in lung cancer

**Fig. 8:**
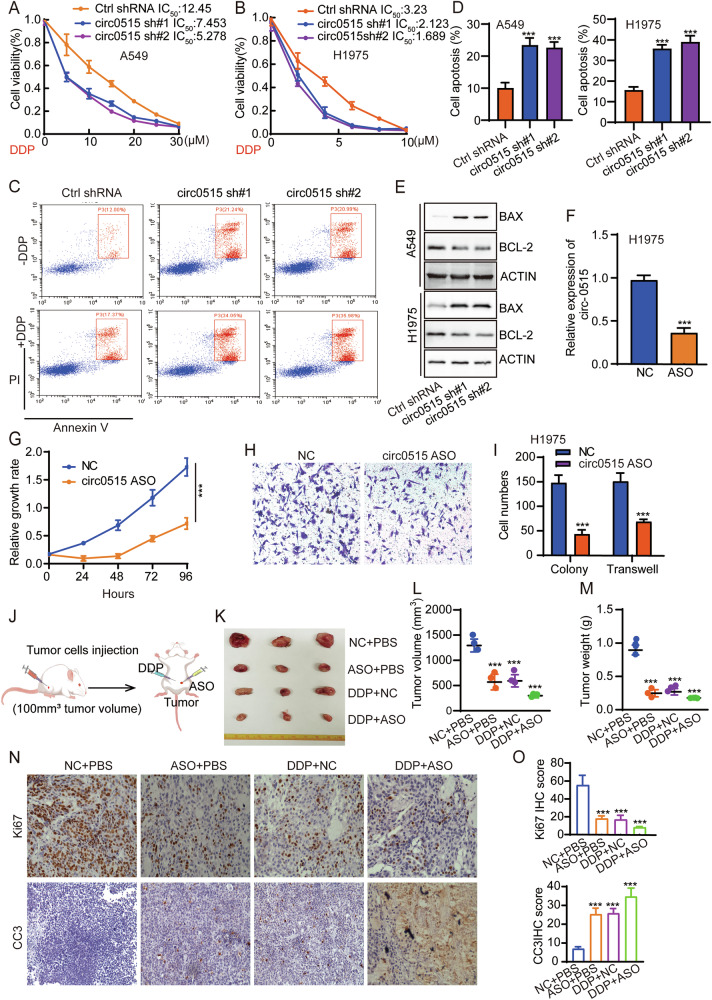
Circ0515 ASO reverses cisplatin resistance in lung cancer cells. **A**, **B** The effects of circ0515 knockdown on cisplatin resistance in lung cancer cells were assessed using the CCK8 assay. **C**, **D** Flow cytometry was used to evaluate the impact of circ0515 knockdown on cisplatin resistance in lung cancer cells. **E** Western blot experiments detect the effect of circ0515 knockdown on cell apoptosis-related proteins in lung cancer cells. **F** qPCR experiments were performed to assess the efficiency of circ0515 ASO on H1975 lung cancer cells. **G** CCK8 experiments detect the effect of circ0515 ASO on lung cancer cell proliferation. **H**, **I** Clonogenic assays and transwell assays detect the effect of circ0515 ASO on lung cancer cell proliferation and migration abilities. **J**–**M** Xenograft experiments in nude mice to assess the impact of the circ0515 ASO and DDP on lung cancer tumor growth. **M** shows the tumor weight, L shows the tumor volume. (*n* = 3 mice per group) **N**, **O** IHC experiments to detect the expression of Ki67, and CC3 in the aforementioned tumor tissues. sh#1 circ0515 shRNA#1, sh#2 circ0515 shRNA#2. NC negative control, ASO circ0515 ASO, DDP cisplatin. All data and error bars are presented as the mean ± SDs. *P < 0.05, **P < 0.01, ***P < 0.001 (Student’s *t*-test).

Cisplatin is a commonly used chemotherapy drug for lung cancer, but resistance remains a significant challenge in treatment [38]. Studies have shown that mitochondrial oxidative phosphorylation (OXPHOS) is closely related to cisplatin resistance. Lung cancer cells may enhance OXPHOS to meet the metabolic demands, and cells with enhanced OXPHOS may possess stronger antioxidant capabilities, reducing cisplatin-induced ROS damage and promoting resistance [39]. Cisplatin-resistant cells may rely on OXPHOS to maintain survival and adapt to drug pressure. Enhanced OXPHOS may regulate Bcl-2 family proteins to reduce cisplatin-induced apoptosis [40]. Based on these reports, we hypothesize that circ0515 may regulate mitochondrial oxidative phosphorylation and the electron transport chain, thus affecting cisplatin resistance in lung cancer. To further verify this hypothesis, CCK-8 assays showed that knockdown of circ0515 significantly enhanced lung cancer cell sensitivity to cisplatin, inhibiting lung cancer cell proliferation (Fig. 8A, B). Flow cytometry analysis further revealed that circ0515 knockdown increased DDP sensitivity, significantly enhancing lung cancer cell apoptosis (Fig. 8C, D). Western blot analysis showed that silencing circ0515 upregulated pro-apoptotic protein BAX expression while downregulating anti-apoptotic protein BCL-2 expression (Fig. 8E and Fig. S6D, S6E). Antisense oligonucleotides (ASOs), a promising small-molecule nucleic acid therapy for cancer, were designed to specifically target circ0515 (Fig. 8F). In vitro, ASO significantly inhibited lung cancer cell proliferation and migration compared to controls (Fig. 8G–I). Since circ0515 regulates DDP sensitivity, we hypothesized targeting circ0515 with ASO reverses cisplatin resistance in lung cancer. Based on this hypothesis, in vivo mouse xenograft tumor model experiments demonstrated that ASO and DDP alone inhibited tumor growth, while their combination significantly enhanced the suppression of tumor growth, as evidenced by reductions in tumor volume and weight (Fig. 8J–M). Immunohistochemical analysis further revealed that the combined treatment with ASO and DDP significantly downregulated KI67 expression and upregulated CC3 expression (Fig. 8N, O). These results suggest that circ0515 plays a crucial role in the progression of lung adenocarcinoma and cisplatin resistance.

## Discussion

Circular RNAs (circRNAs) are a class of endogenous and conserved non-coding RNAs characterized by a covalently closed circular structure formed through back-splicing, which lacks both 3’ and 5’ ends [6, 41]. Compared to most other linear RNAs, circRNAs exhibit higher stability, making them effective biomarkers for cancer diagnosis and treatment [41, 42]. Circ0515, a newly identified circRNA derived primarily from the RPPH1 gene and located at chr14: 20811305-20811534, has been shown to be highly expressed in hepatocellular carcinoma. Knockdown of circ0515 has been demonstrated to inhibit the migration and invasion of hepatocellular carcinoma cells and promote apoptosis [43]. These findings suggest that circ0515 plays an oncogenic role in liver and bladder cancer, yet its biological function in lung cancer remains unclear.

This study is the first to uncover the expression profile and functional mechanisms of circ0515 in lung cancer. We found that circ0515 is significantly upregulated in lung cancer tissues and cells. It functions as a molecular sponge for miRNA-328-3p, reducing the inhibitory effect of miRNA-328-3p on its downstream target gene YWHAZ, leading to the upregulation of YWHAZ. As a known signaling regulator, YWHAZ activates the AKT signaling pathway, promoting cell proliferation and migration. Functional assays further demonstrated that high expression of circ0515 significantly enhances the malignant biological behaviors of lung cancer cells, including proliferation and migration, whereas inhibition of circ0515 expression markedly suppresses these processes.

Our findings suggest that circ0515 exerts oncogenic effects via the “circRNA-miRNA-mRNA” regulatory axis, providing new insights into the mechanisms underlying lung cancer pathogenesis [44]. Specifically, circ0515 promotes lung cancer progression through the miRNA-328-3p/YWHAZ/AKT signaling axis. Notably, the AKT pathway is considered a key oncogenic pathway in various cancers, implying that circ0515 might exhibit similar functions in other cancer types. Additionally, these results provide theoretical support for the potential of circ0515 as a diagnostic biomarker and therapeutic target for lung cancer. However, further in vivo studies and validation with clinical samples are needed to investigate the diagnostic value and feasibility of therapeutic targeting of circ0515 in lung cancer patients.

The PI3K/AKT signaling pathway plays a central role in regulating cell survival, proliferation, and metabolism [45]. Aberrant activation of this pathway is frequently observed in various cancers, including non-small cell lung cancer, and is closely associated with enhanced mitochondrial function and chemoresistance. In addition to PI3K/AKT, other signaling pathways such as AMP-activated protein kinase (AMPK) and hypoxia-inducible factor-1 alpha (HIF-1α) could also play significant roles in mitochondrial homeostasis and drug resistance. AMPK acts as a key energy sensor that promotes mitochondrial biogenesis and oxidative metabolism under metabolic stress, while HIF-1α, often activated in the hypoxic tumor microenvironment, reprograms cellular metabolism toward glycolysis and contributes to resistance to chemotherapy. The interplay between these pathways may orchestrate a complex regulatory network that supports tumor cell survival and adaptation, highlighting their potential as therapeutic targets in overcoming cancer drug resistance. In this study, we found that both the downstream target gene enrichment analysis of miR-328-3p and the analysis of downregulated genes from circ0515/RBM45 transcriptome sequencing commonly highlighted the AKT signaling pathway. Therefore, we focused on investigating the role of the AKT signaling pathway in drug resistance and progression of lung cancer cells. In future studies, we also plan to further explore other signaling pathways, particularly AMPK and HIF-1α, and their critical roles in mitochondrial metabolism and drug resistance.

RNA binding motif protein 45 is an RNA-binding protein that belongs to the RNA-binding protein (RBP) family [46]. RBM45 contains multiple RNA recognition motifs (RRMs), endowing it with high specificity for RNA binding. It plays a critical role in regulating RNA stability and protein translation [47–49]. RBM45 is essential in neurodevelopment and closely associated with neuronal function maintenance [50]. Previous studies have shown that RBM45 participates in neuronal survival and function by regulating RNA metabolism and stability [50]. However, the functional mechanisms of RBM45 in cancer remain largely unexplored. In this study, we identified that circ0515 interacts with RBM45 and colocalizes with it in the cytoplasm. We revealed the collaborative mechanism between circ0515 and RBM45 in lung adenocarcinoma and further clarified their role in promoting cisplatin resistance. Our findings demonstrate that circ0515 directly interacts with the classical RBP RBM45, enhancing RBM45’s ability to bind to the transcript of its downstream target gene SDHB. This interaction significantly stabilizes the SDHB transcript and increases its protein expression. As a key subunit of mitochondrial complex II, SDHB directly regulates mitochondrial oxidative phosphorylation and succinate metabolism. Its upregulation significantly enhances mitochondrial function, meeting the energy demands of rapidly proliferating tumor cells. Moreover, we found that activation of the circ0515/RBM45/SDHB axis increases cisplatin resistance in lung cancer cells, potentially by enhancing oxidative phosphorylation, reducing cisplatin-induced ROS damage, and inhibiting apoptosis. Based on these results, we propose that circ0515 and RBM45 collaboratively promote mitochondrial metabolism and energy generation, conferring greater survival and drug resistance to lung cancer cells. This discovery reveals a novel molecular mechanism of circRNA and RBP in tumor metabolic regulation and provides theoretical support for developing circ0515 and RBM45 as potential therapeutic targets. Particularly in the context of escalating cisplatin resistance, targeting circ0515 or RBM45 may effectively reverse resistance and significantly improve therapeutic outcomes. However, further studies are required to validate the precise role of this axis in mitochondrial metabolism regulation and drug resistance mechanisms and to explore its therapeutic potential in in vivo models.

Although we were the first to identify that circ_0000515 recruits RBM45 to regulate the stability of SDHB transcripts, thereby reshaping mitochondrial metabolism, our study has significant limitations. First, the molecular mechanisms underlying the aberrant upregulation of circ0515 in lung cancer remain unclear. For instance, it is unknown whether its dysregulated expression is influenced by other RNA chemical modifications, such as m6A or m5C. Second, it is uncertain whether the interaction between circ0515 and RBM45 directly regulates the translation of downstream target genes, rather than acting at the post-transcriptional level, in different disease contexts. Finally, whether the circ0515/RBM45/SDHB signaling axis functions broadly across various cancers or represents a lung cancer-specific mechanism remains unresolved. These unanswered questions warrant further in-depth investigation in future studies.

## Conclusions

In summary, this study systematically reveals the critical role of circ0515 in lung cancer progression and cisplatin resistance. We demonstrate that circ0515 is significantly upregulated in lung cancer and promotes tumor malignancy through multilayered molecular mechanisms. On one hand, circ0515 acts as a molecular sponge for miRNA-328-3p, upregulating the downstream target gene YWHAZ, activating the AKT signaling pathway, and significantly enhancing the proliferation and migration of lung cancer cells. On the other hand, circ0515 synergizes with RBM45 to maintain the stability of the SDHB transcript, increase SDHB protein expression, promote mitochondrial oxidative phosphorylation and succinate metabolism, and ultimately enhance cisplatin resistance in lung cancer cells. These findings provide new insights into the molecular mechanisms of circ0515 in lung cancer progression and offer theoretical support for its potential as a therapeutic target for lung cancer. Future studies should further explore the clinical feasibility of circ0515, including its value in lung cancer diagnosis and targeted therapy.

## Supplementary information

original data

Circ0515 reprogramming mitochondrial succinate metabolism and promotes lung adenocarcinoma progression through regulating SDHB

## Data Availability

The datasets used and/or analyzed during the current study are available from the corresponding author upon reasonable request.
