# Correction: Circ0515 reprogramming mitochondrial succinate metabolism and promotes lung adenocarcinoma progression through regulating SDHB

**DOI:** 10.1038/s41419-026-08999-1

**Published:** 2026-07-23

**Authors:** Yixiao Yuan, Yue Wu, Chunhong Li, Zuotian Huang, Dadi Peng, Zhongjun Wu, xiulin jiang

**Affiliations:** 1 The First Affiliated Hospital of Chongqing Medical University, Chongqing, China; 2 Department of Medicine, UF Health Cancer Center, University of Florida, Gainesville, FL USA; 3 Department of Oncology, Suining Central Hospital, Suining, Sichuan China; 4 Chongqing University Cancer Hospital, Chongqing, China

**Keywords:** Cancer epigenetics, Diagnostic markers

Correction to: *Cell Death & Disease* 10.1038/s41419-025-07830-7, published online 05 July 2025

During a post-publication review of the published article, we identified that incorrect Western blot images for mTOR and AKT were inadvertently included in Figure 4K. This error occurred during the final assembly of the figure due to an oversight and mismanagement of image files. The original experimental data have been fully preserved and carefully rechecked. The corrected images are authentic and correspond to the original experiments. Importantly, this correction does not alter the results, data interpretation, conclusions, or any descriptions in the article. Therefore, a correction is required to replace the incorrect images in Figure 4K and ensure the accuracy and integrity of the published record.

**Original figure 4K** 
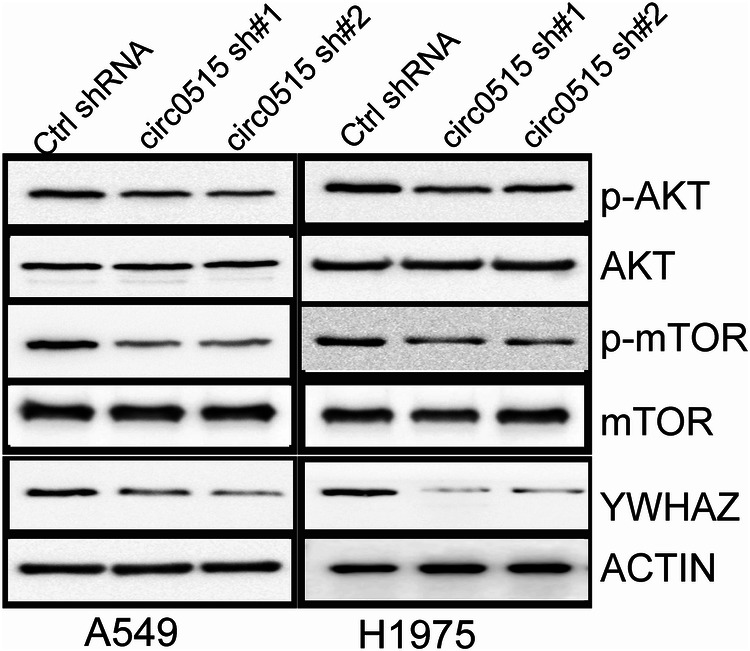


**Amended figure 4K** 
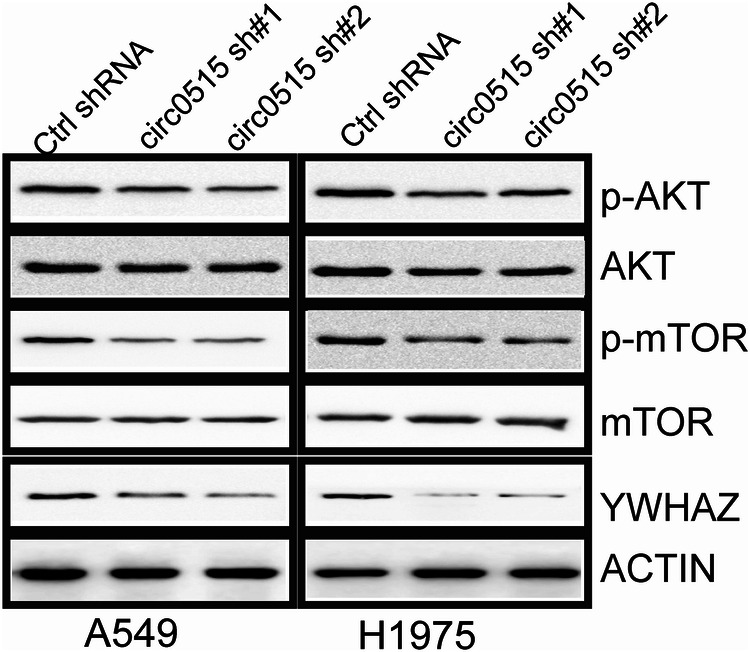


The original article has been corrected.

