# Supplementary material for: Circ0515 reprogramming mitochondrial succinate metabolism and promotes lung adenocarcinoma progression through regulating SDHB
Source: Cell Death Dis. 2025 Jul 5;16(1):497. doi: 10.1038/s41419-025-07830-7 (PMC12228733; doi:10.1038/s41419-025-07830-7)

Full and uncropped western blots

Figure 2I Left

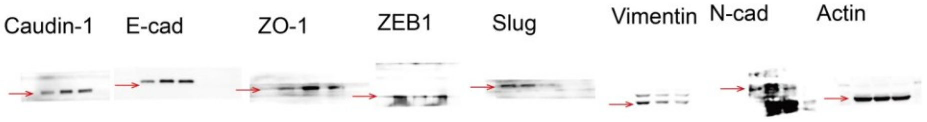

Figure 2I Right

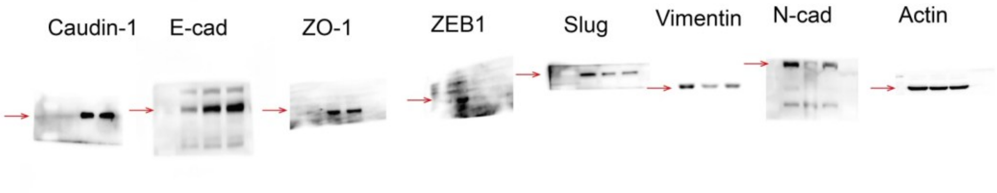

Figure 4K

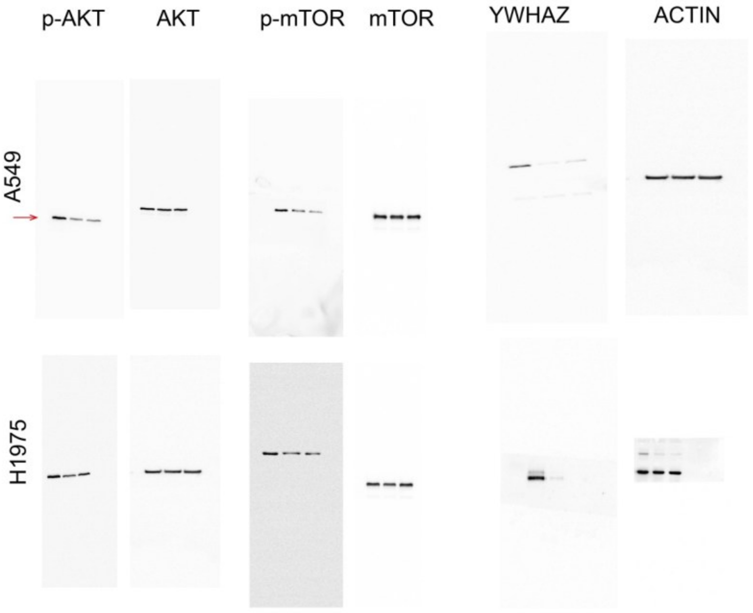

Figure 5C-5D

5C

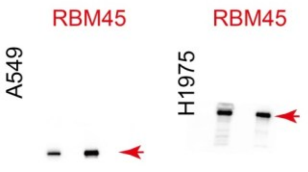

5D

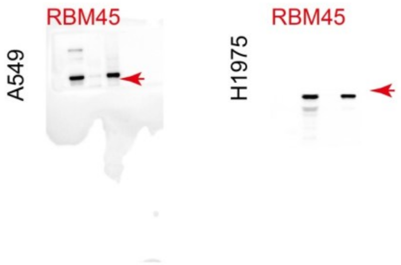

Figure 5J

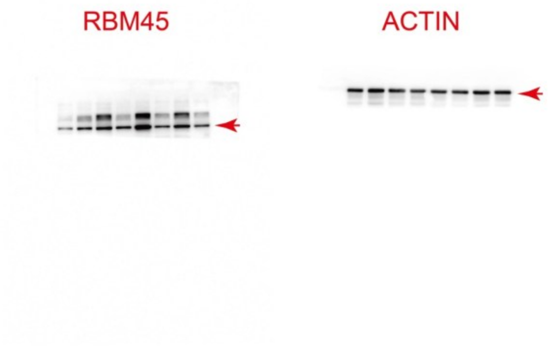

Figure 6C

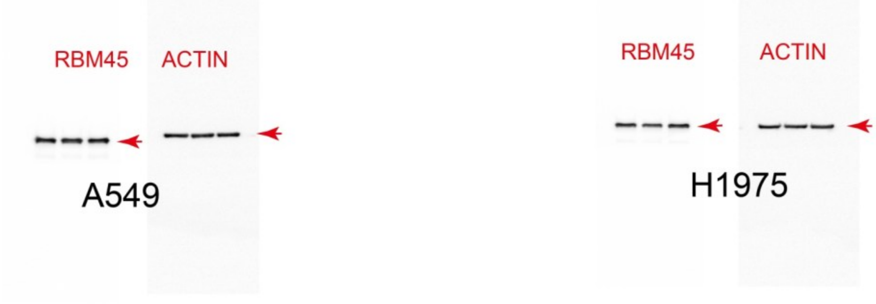

Figure S5H

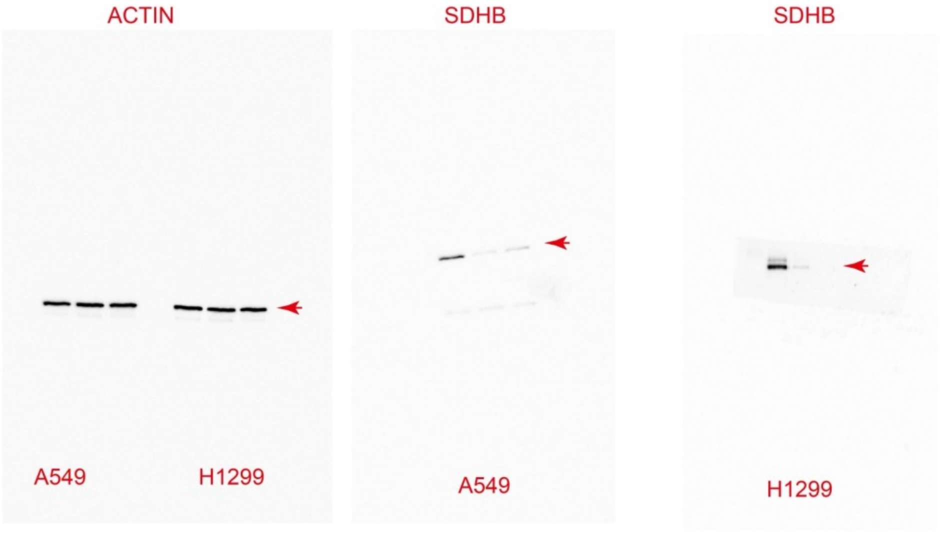

Figure 8E

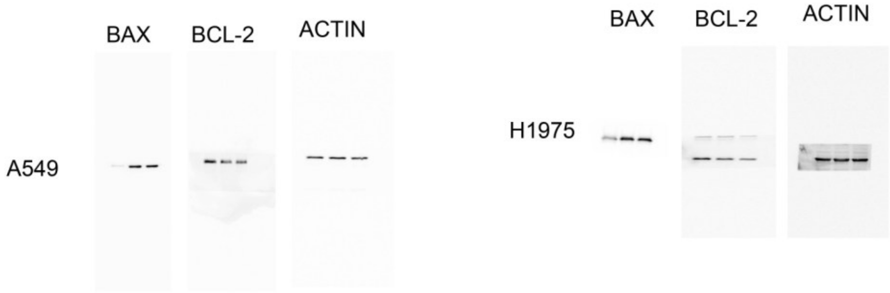

Supplement: Supplementary file 1 — original data [file 41419_2025_7830_MOESM1_ESM.pdf]
