# Supplementary material for: Circ0515 reprogramming mitochondrial succinate metabolism and promotes lung adenocarcinoma progression through regulating SDHB
Source: Cell Death Dis. 2025 Jul 5;16(1):497. doi: 10.1038/s41419-025-07830-7 (PMC12228733; doi:10.1038/s41419-025-07830-7)
Supplement: Supplementary file 2 — Circ0515 reprogramming mitochondrial succinate metabolism and promotes lung adenocarcinoma progression through regulating SDHB [file 41419_2025_7830_MOESM2_ESM.docx]

**Circ0515 reprogramming mitochondrial succinate metabolism and promotes lung adenocarcinoma progression through regulating SDHB**

Yixiao Yuan ^1,4#^, Yue Wu^1#^, Chunhong Li ^1,2#^, Zuotian Huang^3^, Dadi Peng^1^, Zhongjun Wu^1*^, Xiulin Jiang^4*^,

1. The First Affiliated Hospital of Chongqing Medical University, Chongqing, 400016, China.
2. Department of Oncology Suining Central Hospital Suining Sichuan China.

3、Chongqing University Cancer Hospital, Chongqing 400030, China

4、Department of Medicine, UF Health Cancer Center, University of Florida, Gainesville, FL 32610, USA

^#^Contributed equally

^*^Corresponding author:

Xiulin Jiang:xiulin.jiang@ufl.edu

Zhongjun Wu:wzjtcy@126.com

This file includes:

1. Figure S1-S6.
2. Tables S1 to S3


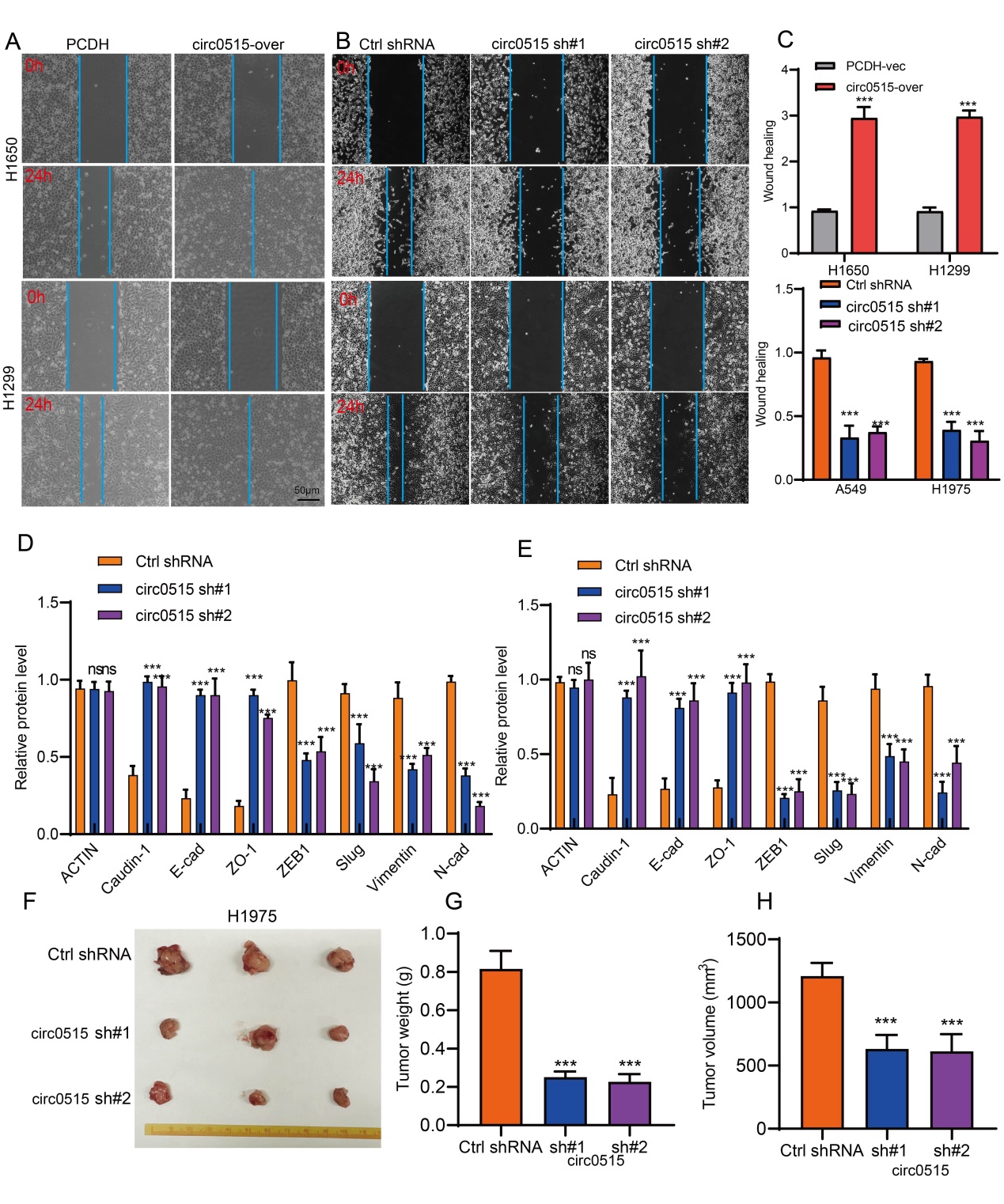


**Figure S1. Knockdown of circ_0515 suppresses lung cancer cell proliferation and migration.**

(A-C) Woundhealing assays detect the effect of circ0515 knockdown and overexpression on lung cancer cell migration ability. (D–E) Quantitative analysis of EMT-related protein expression following circ_0515 knockdown. (F-H) Xenograft experiments in nude mice to assess the impact of the circ0515 knockdown on lung cancer tumor growth. (G) shows the tumor weight, (H) shows the tumor volume, (n = 3 mice per group), PCDH=PCDH-Vec, circ0515 Over= circ0515 Overexpression, sh#1= circ0515 shRNA#1, sh#2= circ0515 shRNA#2. All data and error bars are presented as the mean ± SDs. **P* < 0.05, ***P* < 0.01, ****P* < 0.001 (Student’s *t*-test).


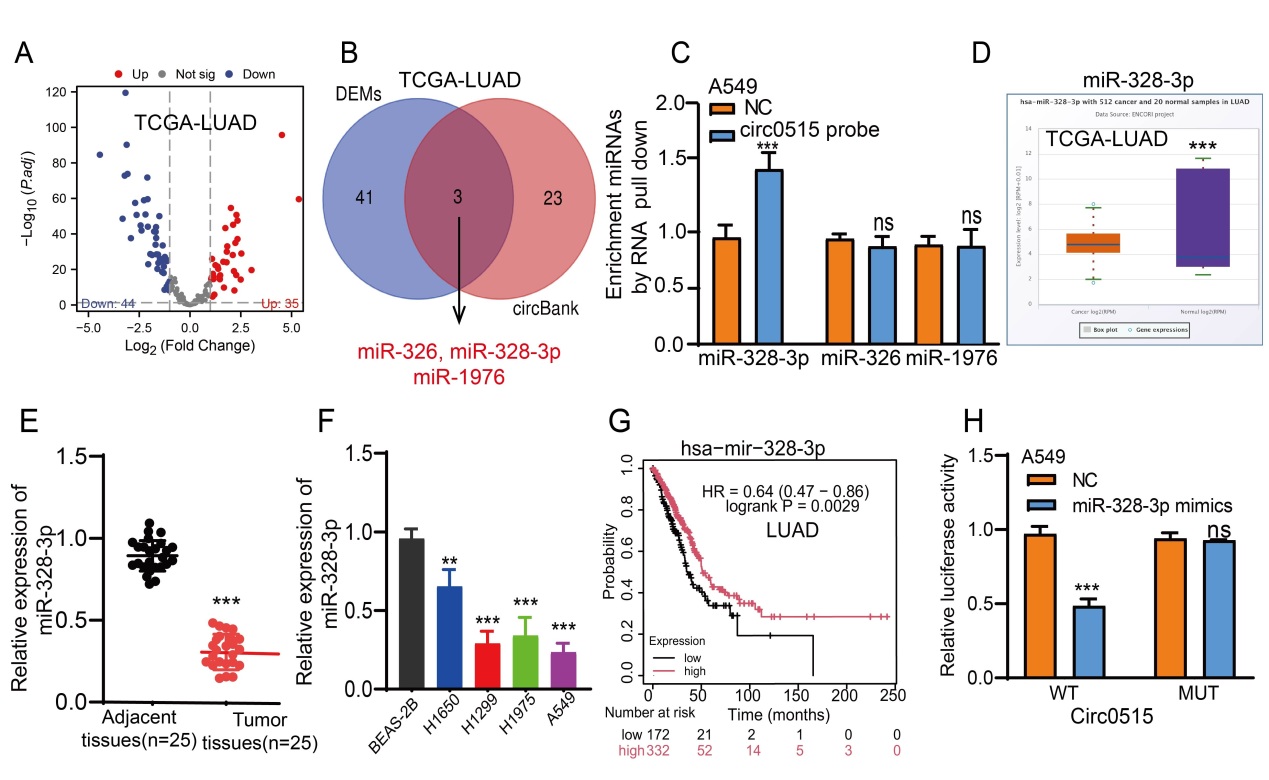


**Figure S2. circ_0515 functions as a molecular sponge for miR-326.**

(A-B) The downregulated miRNAs in the TCGA-LUAD dataset were intersected with miRNAs predicted to bind circ0515 according to the circBank database. (C) RNA pull-down assay validating the interaction between circ0515 and miRNA. (D) The expression levels of miR-328-3p were analyzed using the TCGA-LUAD dataset. (E) qPCR experiments were performed to assess miR-328-3p expression levels in 25 pairs of matched lung cancerous and adjacent non-cancerous tissues. (F) qPCR experiments were conducted to measure the expression levels of miR-328-3p in lung cancer cells compared to normal lung epithelial cells. (G) The prognostic value of miR-328-3p in LUAD was evaluated using the TCGA database. (H) A dual-luciferase reporter assay was used to assess the binding interaction between circ0515 and miR-328-3p. PCDH=PCDH-Vec, circ0515 Over= circ0515 Overexpression, NC=Negative control, mimics= miR-328-3p mimics, **P* < 0.05, ***P* < 0.01, ****P* < 0.001 (Student’s *t*-test).


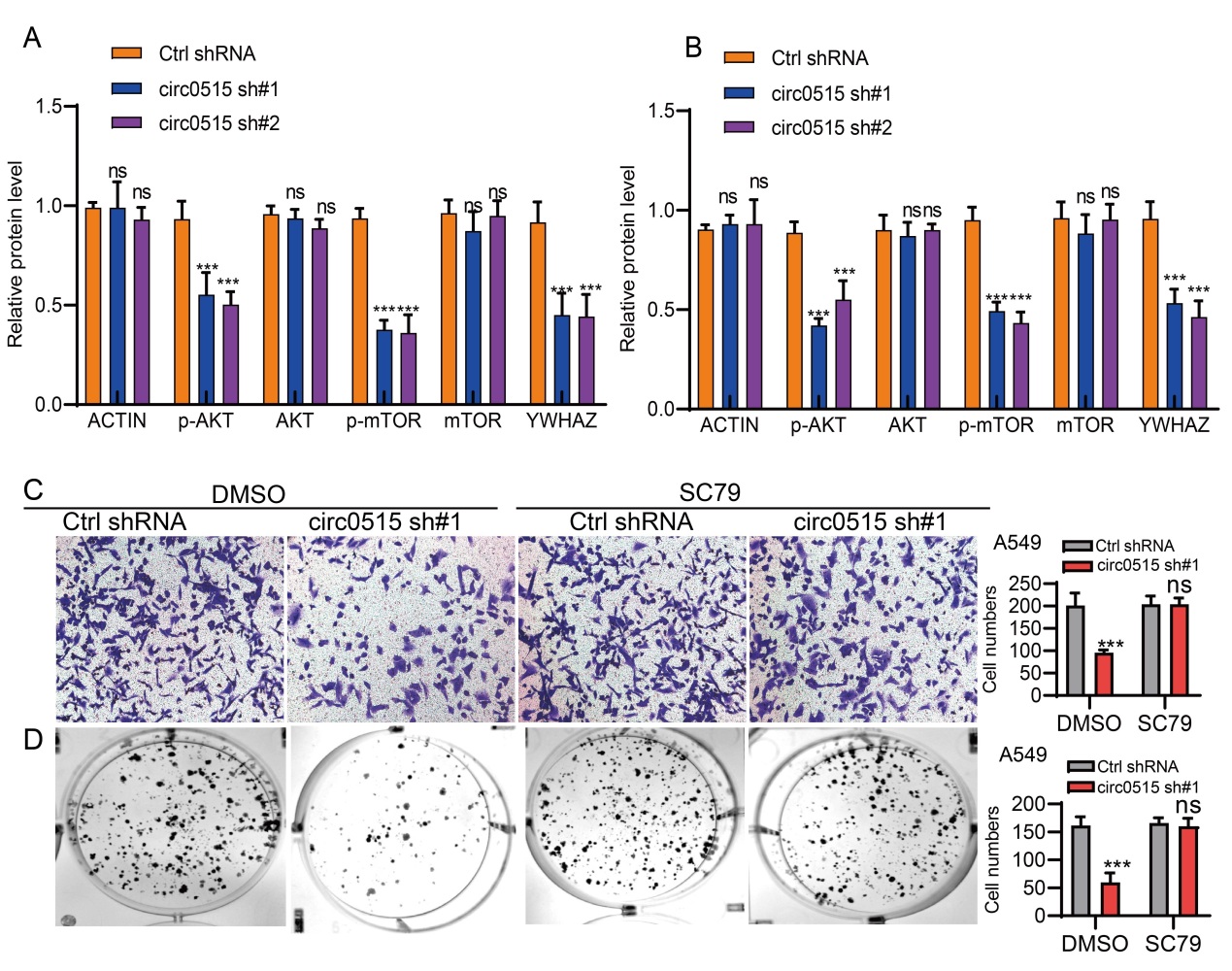


**Figure S3. circ_0515 activates the AKT signaling pathway.**

(A–B) Quantitative data showing reduced expression of AKT pathway-related proteins after circ_0515 knockdown. (C-D) SC79 treatment rescues the inhibited cell migration and proliferation caused by circ0515 knockdown in lung cancer cells. sh#1 = circ0515 shRNA#1, sh#2 = circ0515 shRNA#2. **P* < 0.05, ***P* < 0.01, ****P* < 0.001 (Student’s *t*-test).


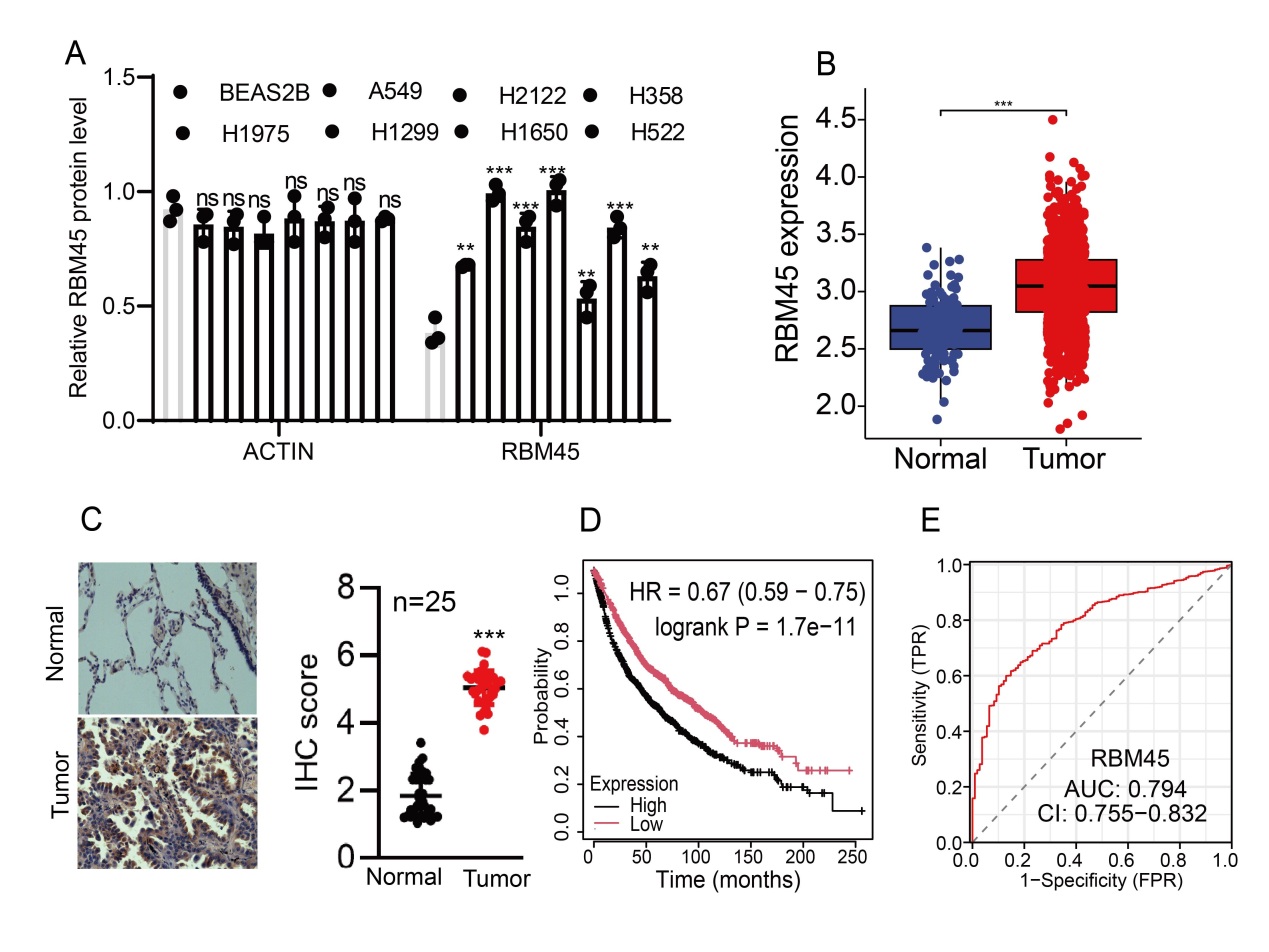


**Figure S4. RBM45 is upregulated in lung cancer tissues.**

(A) Quantification of RBM45 protein expression levels in lung cancer cell lines. (B) TCGA database analysis of RBM45 expression in lung cancer. (C) IHC experiments validating RBM45 expression in lung cancer tissues versus control tissues. n=25. (D) TCGA database analysis of the correlation between RBM45 expression levels and prognosis in lung cancer patients. (E) ROC curve analysis of RBM45 in lung cancer samples. **P* < 0.05, ***P* < 0.01, ****P* < 0.001 (Student’s *t*-test).


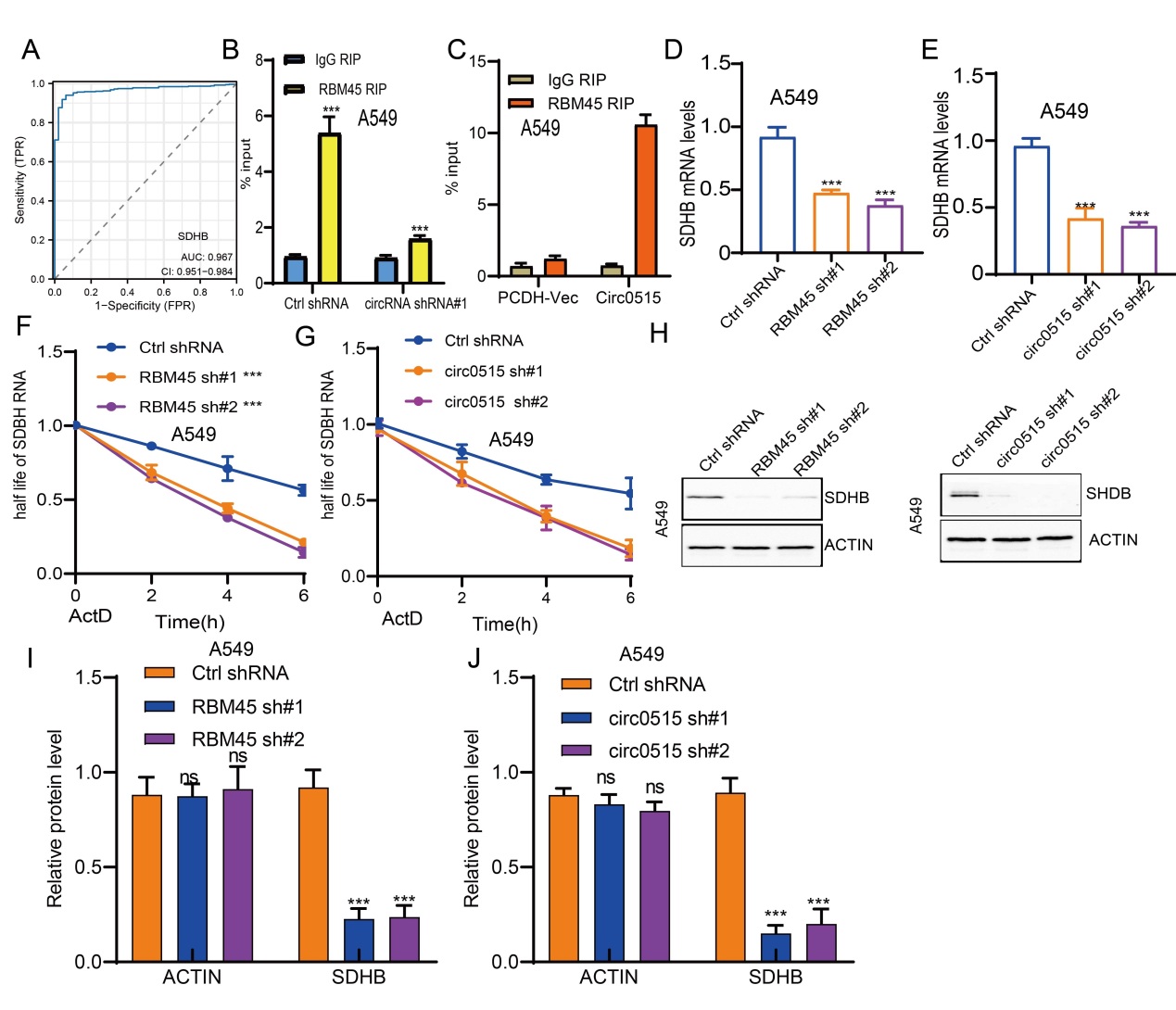


**Figure S5. The circ_0515/RBM45 complex regulates SDHB expression.**

(A) AUC curve analysis of SDHB in LUAD using the TCGA database. (B-C) RIP-qPCR analysis of RBM45 binding to SDHB after circ0515 knockdown or overexpression in A549 cells. (D) qPCR analysis of the effects of RBM45 knockdown on SDHB mRNA expression. (E) qPCR analysis of the effects of circ0515 knockdown on SDHB mRNA expression. (F) RNA half-life assay to assess the effects of RBM45 knockdown on SDHB mRNA stability. (G) RNA half-life assay to assess the effects of circ0515 knockdown on SDHB mRNA stability. Cells were then treated with 5 μg/mL actinomycin D solution and cultured at different time intervals. (H) Western blot analysis of the effects of RBM45 or circ0515 knockdown on SDHB protein levels. (I–J) Quantitative protein data corresponding to panels G–H. **P* < 0.05, ***P* < 0.01, ****P* < 0.001 (Student’s *t*-test).


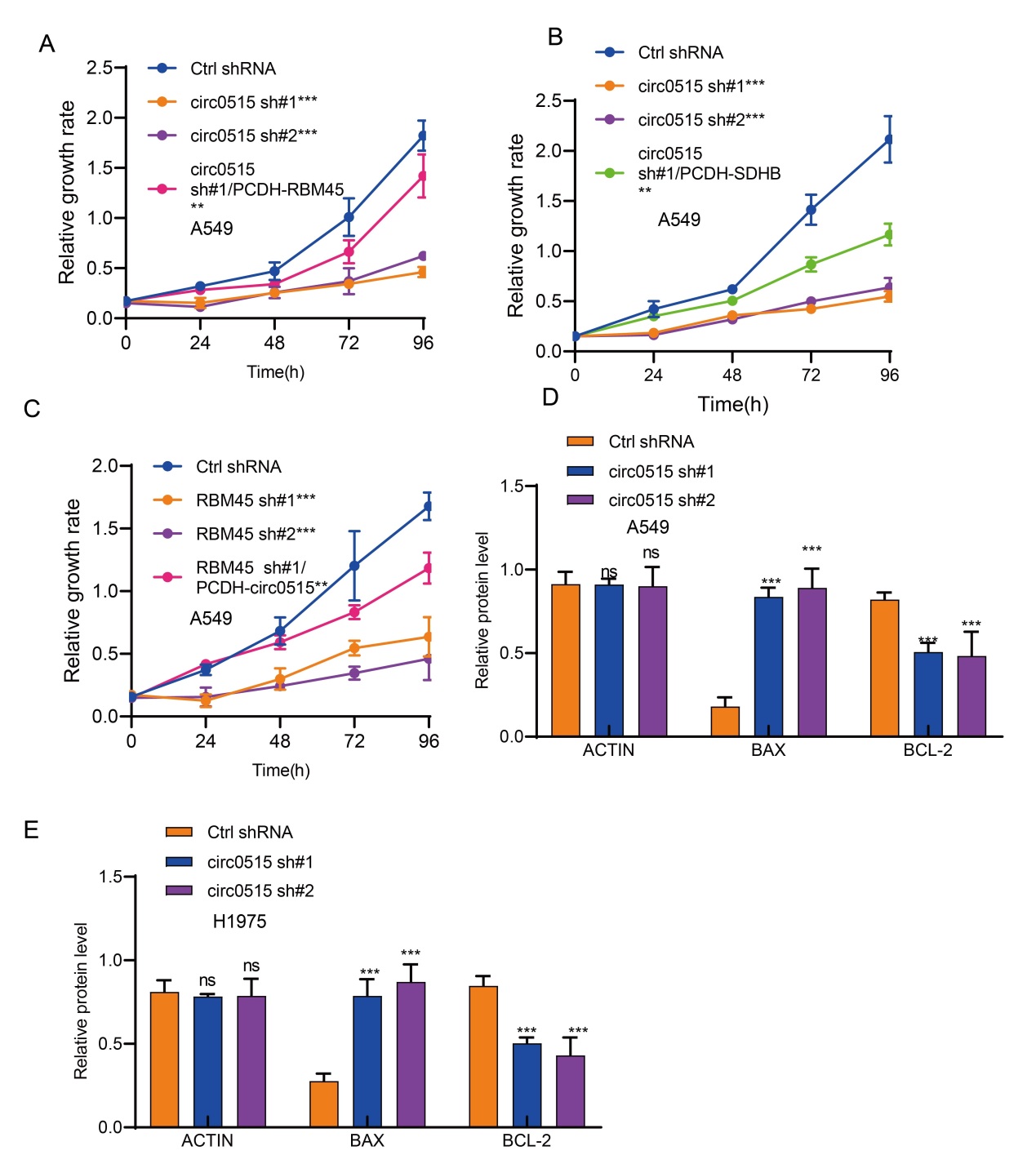


**Figure S6. Knockdown of circ_0515 induces cisplatin resistance in lung cancer cells.**

(A) Overexpression of RBM45 rescues the inhibitory effect of circ_0515 knockdown on lung cancer cell proliferation. (B) Overexpression of SDHB reverses the proliferation suppression induced by circ_0515 knockdown. (C) Overexpression of circ_0515 rescues the proliferation inhibition caused by RBM45 knockdown. (D–E) Quantitative analysis of apoptosis-related protein levels following circ_0515 knockdown. **P* < 0.05, ***P* < 0.01, ****P* < 0.001 (Student’s *t*-test).

**Table S1. ShRNA primer and oligos used in this study.**

| Primer Name | Primer Sequences(5'-3') |
| --- | --- |
| Human β-actin_F | AAGTGTGACGTGGACATCCGC |
| Human β-actin_R | CCGGACTCGTCATACTCCTGCT |
| Human circ_0000515-F | GGTCAGACTGGGCAGGAGAT |
| Human circ_0000515-R | GAGTGACAGGACGCACTCAG |
| Human miR-328-3p _F | ATATCTGGCCCTCTCTGCCC |
| Human miR-328-3p _R | GTGCAGGGTCCGAGGT |
| Human YWHAZ _F | CCTGCATGAAGTCTGTAACTGAG |
| Human YWHAZ _R | GACCTACGGGCTCCTACAACA |
| Human EIF4EBP1 _F | CTATGACCGGAAATTCCTGATGG |
| Human EIF4EBP1 _R | CCCGCTTATCTTCTGGGCTA |
| Human ITGA5 _F | GGCTTCAACTTAGACGCGGAG |
| Human ITGA5 _R | TGGCTGGTATTAGCCTTGGGT |
| Human PPP2R5D _F | ACTTCGTGTCAGACCCACTCA |
| Human PPP2R5D _R | CTCAGGGTAAATGGCCTCAGT |
| Human circ_0000515 shRNA #1 | GAGGTGAGTTCCCAGAGAA |
| Human circ_0000515 shRNA #2 | CCGGAGCTTGGAACAGACT |
| Human U6 _F | CTCGCTTCGGCAGCACA |
| Human U6 _R | AACGCTTCACGAATTTGCGT |
| Human RBM45_F | TCAGCAAGTACACACCTGAGT |
| Human RBM45_R | AGATGATCGGGACTGAGCAAT |
| Human SDHB-F | ACCTTCCGAAGATCATGCAGA |
| Human SDHB-R | GTGCAAGCTAGAGTGTTGCCT |

**Table S2. Antibodies and oligos used in this study.**

| **Antibody Name** | **Catalog Number** | **Dilution** | **Supplier** | **Species** |
| --- | --- | --- | --- | --- |
| E-cadherin | ab40772 | 1:500 | abcam | Rabbit |
| N-cadherin | ab18203 | 1:1000 | abcam | Rabbit |
| Vimentin | 103661-1-AP | 1:1000 | Proteintech | Rabbit |
| β-actin | 60008-1-1g | 1:5000 | Proteintech | Mouse |
| Akt | 9272 | 1:1000 | CST | Rabbit |
| p-Akt | 9271 | 1:1000 | CST | Rabbit |
| GSK3β | 5676 | 1:1000 | CST | Rabbit |
| p-GSK3β | 9336 | 1:1000 | CST | Rabbit |
| S6K | 9202 | 1:1000 | CST | Rabbit |
| p-S6K | 9205 | 1:1000 | CST | Rabbit |
| mTOR | 2983 | 1:2000 | CST | Rabbit |
| p-mTOR | 5536 | 1:2000 | CST | Rabbit |
| Ki67 | Kit-0005 | 1:1000 | MXB | Mouse/Rabbit |
| RBM45 | HPA020448 | 1:1000 | Sigma | Rabbit |
| SDHB | 92649 | 1:1000 | CST | Rabbit |
| BAX | 2772 | 1:1000 | CST | Rabbit |
| BCL-2 | 15071 | 1:1000 | CST | Mouse |

**Table S3. GSE158695 dataset analysis of differentially expressed circRNAs in NSCLC.**

| **circRNA** | **Alias** | **P-value** | **FDR** | **FC (abs)** | **Regulate** | **circRNA_type** | **chrom** |
| --- | --- | --- | --- | --- | --- | --- | --- |
| hsa_circRNA_000585 | hsa_circ_0000515 | 0.0002995429 | 0.00124763 | 7.461476 | up | intragenic | chr14 |
| hsa_circRNA_100945 | hsa_circ_0008602 | 2.73906E-05 | 0.047775 | 0.422342 | down | exonic | chr11 |
| hsa_circRNA_103444 | hsa_circ_0008797 | 6.48947E-05 | 0.047775 | 0.0834 | down | exonic | chr3 |
| hsa_circRNA_103082 | hsa_circ_0060828 | 9.33437E-05 | 0.047775 | 0.220565 | down | exonic | chr20 |
| hsa_circRNA_104513 | hsa_circ_0007518 | 9.84534E-05 | 0.047775 | 0.142566 | down | exonic | chr7 |
| hsa_circRNA_104126 | hsa_circ_0076798 | 0.000155828 | 0.060492 | 0.076498 | down | exonic | chr6 |
| hsa_circRNA_100832 | hsa_circ_0022378 | 0.000279794 | 0.066747 | 0.244314 | down | exonic | chr11 |
| hsa_circRNA_100989 | hsa_circ_0000370 | 0.000291582 | 0.066747 | 0.197736 | down | exonic | chr11 |
| hsa_circRNA_100160 | hsa_circ_0011536 | 0.000297608 | 0.066747 | 0.063069 | down | exonic | chr1 |
| hsa_circRNA_000911 | hsa_circ_0001184 | 0.000356759 | 0.066747 | 0.097101 | down | intronic | chr21 |
| hsa_circRNA_101491 | hsa_circ_0034762 | 0.000374777 | 0.066747 | 0.10373 | down | exonic | chr15 |
| hsa_circRNA_100891 | hsa_circ_0023685 | 0.000378269 | 0.066747 | 0.147272 | down | exonic | chr11 |
| hsa_circRNA_000031 | hsa_circ_0000009 | 0.000489049 | 0.069163 | 0.170255 | down | intronic | chr1 |
| hsa_circRNA_002086 | hsa_circ_0001693 | 0.000498859 | 0.069163 | 0.099202 | down | exonic | chr7 |
| hsa_circRNA_102774 | hsa_circ_0055412 | 0.00065685 | 0.084996 | 0.108804 | down | exonic | chr2 |
| hsa_circRNA_105034 | hsa_circ_0001947 | 0.000790063 | 0.090207 | 0.34135 | down | exonic | chrX |
| hsa_circRNA_104016 | hsa_circ_0075048 | 0.000959372 | 0.093499 | 3.285596 | up | exonic | chr5 |
| hsa_circRNA_001846 | hsa_circ_0000520 | 0.001360196 | 0.112137 | 4.274055 | up | intragenic | chr14 |
| hsa_circRNA_104310 | hsa_circ_0079385 | 0.001483888 | 0.115209 | 0.056023 | down | exonic | chr7 |
| hsa_circRNA_102239 | hsa_circ_0000817 | 0.001801393 | 0.120158 | 0.165218 | down | exonic | chr17 |
| hsa_circRNA_000167 | hsa_circ_0000518 | 0.001829074 | 0.120158 | 3.923004 | up | intragenic | chr14 |
| hsa_circRNA_102509 | hsa_circ_0006446 | 0.00184126 | 0.120158 | 0.133131 | down | exonic | chr19 |
| hsa_circRNA_100259 | hsa_circ_0006677 | 0.001891032 | 0.120158 | 0.204744 | down | exonic | chr1 |
| hsa_circRNA_101969 | hsa_circ_0041821 | 0.001944708 | 0.120158 | 0.250138 | down | exonic | chr17 |
| hsa_circRNA_100900 | hsa_circ_0002476 | 0.002042879 | 0.120158 | 2.762078 | up | exonic | chr11 |
| hsa_circRNA_101338 | hsa_circ_0031570 | 0.002208234 | 0.124763 | 2.273821 | up | exonic | chr14 |
| hsa_circRNA_104099 | hsa_circ_0076092 | 0.00254847 | 0.124763 | 0.083749 | down | exonic | chr6 |
| hsa_circRNA_101958 | hsa_circ_0041555 | 0.002716626 | 0.124763 | 0.11361 | down | exonic | chr17 |
| hsa_circRNA_104855 | hsa_circ_0087888 | 0.002813812 | 0.124763 | 0.380515 | down | exonic | chr9 |
| hsa_circRNA_102442 | hsa_circ_0049271 | 0.002940841 | 0.124763 | 0.187472 | down | exonic | chr19 |
| hsa_circRNA_001654 | hsa_circ_0001605 | 0.002968725 | 0.124763 | 0.190009 | down | intronic | chr6 |
| hsa_circRNA_100988 | hsa_circ_0000369 | 0.002995433 | 0.124763 | 0.205547 | down | exonic | chr11 |
| hsa_circRNA_000881 | hsa_circ_0000788 | 0.003278866 | 0.124763 | 0.103445 | down | intronic | chr17 |
| hsa_circRNA_104161 | hsa_circ_0077514 | 0.003381951 | 0.124763 | 0.209758 | down | exonic | chr6 |
| hsa_circRNA_104193 | hsa_circ_0077930 | 0.003406705 | 0.124763 | 0.123507 | down | exonic | chr6 |
| hsa_circRNA_101282 | hsa_circ_0030569 | 0.003509873 | 0.12616 | 0.140422 | down | exonic | chr13 |
| hsa_circRNA_102078 | hsa_circ_0043893 | 0.003812066 | 0.132384 | 2.047286 | up | exonic | chr17 |
| hsa_circRNA_100202 | hsa_circ_0012185 | 0.004137387 | 0.133342 | 0.272262 | down | exonic | chr1 |
| hsa_circRNA_101213 | hsa_circ_0029426 | 0.004235755 | 0.133342 | 0.242738 | down | exonic | chr12 |
| hsa_circRNA_101066 | hsa_circ_0026337 | 0.004289452 | 0.133342 | 0.159939 | down | exonic | chr12 |
| hsa_circRNA_101957 | hsa_circ_0003239 | 0.004310241 | 0.133342 | 0.120143 | down | exonic | chr17 |
| hsa_circRNA_104670 | hsa_circ_0001818 | 0.00524003 | 0.151804 | 0.066614 | down | exonic | chr8 |
| hsa_circRNA_104415 | hsa_circ_0080813 | 0.00583548 | 0.157315 | 2.127703 | up | exonic | chr7 |
| hsa_circRNA_102535 | hsa_circ_0008354 | 0.006561115 | 0.167567 | 0.156162 | down | exonic | chr19 |
| hsa_circRNA_000486 | hsa_circ_0001853 | 0.006909803 | 0.169434 | 0.134303 | down | antisense | chr9 |
| hsa_circRNA_102741 | hsa_circ_0003497 | 0.006983374 | 0.169434 | 0.242554 | down | exonic | chr2 |
| hsa_circRNA_000104 | hsa_circ_0000266 | 0.008461418 | 0.184535 | 0.135652 | down | intronic | chr10 |
| hsa_circRNA_102744 | hsa_circ_0006037 | 0.00908552 | 0.190825 | 2.144541 | up | exonic | chr2 |
| hsa_circRNA_103237 | hsa_circ_0063526 | 0.00937111 | 0.190825 | 2.540919 | up | exonic | chr22 |
| hsa_circRNA_102445 | hsa_circ_0004552 | 0.010009767 | 0.190825 | 0.145771 | down | exonic | chr19 |
| hsa_circRNA_104881 | hsa_circ_0088088 | 0.010764468 | 0.190825 | 0.128809 | down | exonic | chr9 |
| hsa_circRNA_103211 | hsa_circ_0063158 | 0.010894751 | 0.190825 | 0.146075 | down | exonic | chr22 |
| hsa_circRNA_102171 | hsa_circ_0004789 | 0.011039143 | 0.190825 | 0.093129 | down | exonic | chr17 |
| hsa_circRNA_102012 | hsa_circ_0006686 | 0.011158054 | 0.190825 | 2.711951 | up | exonic | chr17 |
| hsa_circRNA_103415 | hsa_circ_0008234 | 0.01132091 | 0.190825 | 0.404566 | down | exonic | chr3 |
| hsa_circRNA_104882 | hsa_circ_0003597 | 0.011560571 | 0.190825 | 0.144473 | down | exonic | chr9 |
| hsa_circRNA_101937 | hsa_circ_0002637 | 0.012722932 | 0.201466 | 0.479758 | down | exonic | chr17 |
| hsa_circRNA_000792 | hsa_circ_0000291 | 0.012974361 | 0.201466 | 2.142966 | up | intronic | chr11 |
| hsa_circRNA_101722 | hsa_circ_0038111 | 0.013772444 | 0.207766 | 0.243055 | down | exonic | chr16 |
| hsa_circRNA_100292 | hsa_circ_0013353 | 0.014193835 | 0.207766 | 2.787881 | up | exonic | chr1 |
| hsa_circRNA_100048 | hsa_circ_0009715 | 0.014589857 | 0.207766 | 0.285775 | down | exonic | chr1 |
| hsa_circRNA_104858 | hsa_circ_0087897 | 0.014876467 | 0.207766 | 0.128284 | down | exonic | chr9 |
| hsa_circRNA_001678 | hsa_circ_0000517 | 0.015234033 | 0.209563 | 6.343662 | up | intragenic | chr14 |
| hsa_circRNA_104803 | hsa_circ_0087354 | 0.016514243 | 0.214396 | 0.266408 | down | exonic | chr9 |
| hsa_circRNA_000855 | hsa_circ_0000632 | 0.016980606 | 0.218154 | 0.451076 | down | intragenic | chr15 |
| hsa_circRNA_102217 | hsa_circ_0000813 | 0.017249391 | 0.218154 | 0.115593 | down | exonic | chr17 |
| hsa_circRNA_002144 | hsa_circ_0000511 | 0.017417385 | 0.218154 | 2.489903 | up | intragenic | chr14 |
| hsa_circRNA_104936 | hsa_circ_0089033 | 0.017874192 | 0.219749 | 2.29263 | up | exonic | chr9 |
| hsa_circRNA_100751 | hsa_circ_0020934 | 0.018160813 | 0.219749 | 2.343024 | up | exonic | chr11 |
| hsa_circRNA_100899 | hsa_circ_0023736 | 0.018284523 | 0.219749 | 2.293217 | up | exonic | chr11 |
| hsa_circRNA_001264 | hsa_circ_0000086 | 0.018393907 | 0.219749 | 0.28458 | down | antisense | chr1 |
| hsa_circRNA_101888 | hsa_circ_0003026 | 0.018504416 | 0.219749 | 2.149291 | up | exonic | chr16 |
| hsa_circRNA_100395 | hsa_circ_0015278 | 0.019873848 | 0.224065 | 0.122299 | down | exonic | chr1 |
| hsa_circRNA_102046 | hsa_circ_0043256 | 0.019951789 | 0.224065 | 0.101189 | down | exonic | chr17 |
| hsa_circRNA_103929 | hsa_circ_0073736 | 0.020079873 | 0.224065 | 2.404124 | up | exonic | chr5 |
| hsa_circRNA_102929 | hsa_circ_0058497 | 0.021425948 | 0.229363 | 0.3158 | down | exonic | chr2 |
| hsa_circRNA_103162 | hsa_circ_0062317 | 0.021670383 | 0.229848 | 0.15059 | down | exonic | chr22 |
| hsa_circRNA_103456 | hsa_circ_0067127 | 0.0223704 | 0.234708 | 0.131122 | down | exonic | chr3 |
| hsa_circRNA_101458 | hsa_circ_0034044 | 0.022585555 | 0.23506 | 0.265558 | down | exonic | chr15 |
| hsa_circRNA_101033 | hsa_circ_0000384 | 0.02406757 | 0.236766 | 0.489041 | down | exonic | chr12 |
| hsa_circRNA_002178 | hsa_circ_0000519 | 0.024433028 | 0.236766 | 3.037257 | up | intragenic | chr14 |
| hsa_circRNA_101023 | hsa_circ_0004426 | 0.024434964 | 0.236766 | 2.520277 | up | exonic | chr12 |
| hsa_circRNA_001950 | hsa_circ_0000273 | 0.024499533 | 0.236766 | 0.183697 | down | intronic | chr11 |
| hsa_circRNA_101727 | hsa_circ_0000679 | 0.024562884 | 0.236766 | 0.178423 | down | exonic | chr16 |
| hsa_circRNA_103723 | hsa_circ_0070659 | 0.024640245 | 0.236766 | 0.052416 | down | exonic | chr4 |
| hsa_circRNA_101369 | hsa_circ_0032253 | 0.024879811 | 0.237185 | 2.719514 | up | exonic | chr14 |
| hsa_circRNA_101610 | hsa_circ_0003620 | 0.026767663 | 0.249257 | 0.303608 | down | exonic | chr15 |
| hsa_circRNA_103137 | hsa_circ_0061817 | 0.026773209 | 0.249257 | 0.127632 | down | exonic | chr21 |
| hsa_circRNA_103361 | hsa_circ_0001296 | 0.026839102 | 0.249257 | 0.181555 | down | exonic | chr3 |
| hsa_circRNA_001350 | hsa_circ_0000253 | 0.027028098 | 0.249655 | 0.053249 | down | intronic | chr10 |
| hsa_circRNA_102226 | hsa_circ_0046192 | 0.028083017 | 0.254342 | 0.155366 | down | exonic | chr17 |
| hsa_circRNA_100846 | hsa_circ_0022723 | 0.028172907 | 0.254342 | 0.156218 | down | exonic | chr11 |
| hsa_circRNA_102240 | hsa_circ_0046430 | 0.030217356 | 0.260339 | 3.247028 | up | exonic | chr17 |
| hsa_circRNA_104574 | hsa_circ_0083756 | 0.030232487 | 0.260339 | 0.107168 | down | exonic | chr8 |
| hsa_circRNA_101915 | hsa_circ_0041050 | 0.033894239 | 0.268525 | 2.047545 | up | exonic | chr16 |
| hsa_circRNA_001030 | hsa_circ_0000333 | 0.034210411 | 0.268645 | 2.5907 | up | intronic | chr11 |
| hsa_circRNA_101078 | hsa_circ_0026978 | 0.037298807 | 0.273738 | 2.544832 | up | exonic | chr12 |
| hsa_circRNA_000166 | hsa_circ_0000512 | 0.037654792 | 0.273738 | 3.957081 | up | intragenic | chr14 |
| hsa_circRNA_100041 | hsa_circ_0005039 | 0.038619212 | 0.274194 | 0.223063 | down | exonic | chr1 |
| hsa_circRNA_105038 | hsa_circ_0091894 | 0.040093876 | 0.276408 | 2.538437 | up | exonic | chrX |
| hsa_circRNA_102974 | hsa_circ_0001122 | 0.042158906 | 0.283417 | 2.335424 | up | exonic | chr2 |
| hsa_circRNA_001036 | hsa_circ_0000436 | 0.044083521 | 0.283417 | 0.245941 | down | intronic | chr12 |
| hsa_circRNA_102830 | hsa_circ_0001073 | 0.044229937 | 0.283417 | 0.400658 | down | exonic | chr2 |
| hsa_circRNA_102742 | hsa_circ_0001020 | 0.044951804 | 0.283417 | 2.017717 | up | exonic | chr2 |
| hsa_circRNA_103987 | hsa_circ_0004104 | 0.045206561 | 0.283417 | 2.059454 | up | exonic | chr5 |
| hsa_circRNA_100148 | hsa_circ_0011422 | 0.045264913 | 0.283417 | 2.083134 | up | exonic | chr1 |
| hsa_circRNA_101456 | hsa_circ_0006770 | 0.046442062 | 0.287446 | 0.255071 | down | exonic | chr15 |
| hsa_circRNA_104401 | hsa_circ_0005513 | 0.049191568 | 0.297866 | 0.032512 | down | exonic | chr7 |
| hsa_circRNA_103113 | hsa_circ_0005238 | 0.049330865 | 0.297866 | 0.460413 | down | exonic | chr21 |
